# Cellular NS1-BP protein interacts with the mRNA export receptor NXF1 to mediate nuclear export of influenza virus M mRNAs

**DOI:** 10.1016/j.jbc.2024.107871

**Published:** 2024-10-09

**Authors:** Ke Zhang, Tolga Cagatay, Dongqi Xie, Alexia E. Angelos, Serena Cornelius, Vasilisa Aksenova, Sadaf Aslam, Zhiyu He, Matthew Esparza, Ashley Vazhavilla, Mary Dasso, Adolfo García-Sastre, Yi Ren, Beatriz M.A. Fontoura

**Affiliations:** 1 Department of Cell Biology, University of Texas Southwestern Medical Center, Dallas, Texas, USA; 2 Shanghai Institute of Immunity and Infection, Chinese Academy of Sciences, Shanghai, China; 3 Department of Biochemistry, Center for Structural Biology, Vanderbilt University School of Medicine, Nashville, Tennessee, USA; 4 Division of Molecular and Cellular Biology, National Institute of Child Health and Human Development, National Institutes of Health, Bethesda, Maryland, USA; 5 Department of Microbiology, Icahn School of Medicine at Mount Sinai, New York, New York, USA; 6 Global Health and Emerging Pathogens Institute, Icahn School of Medicine at Mount Sinai, New York, New York, USA; 7 Division of Infectious Diseases, Department of Medicine, Icahn School of Medicine at Mount Sinai, New York, New York, USA; 8 Department of Pathology, Molecular and Cell-Based Medicine, Icahn School of Medicine at Mount Sinai, New York, New York, USA; 9 The Tisch Cancer Institute, Icahn School of Medicine at Mount Sinai, New York, New York, USA; 10 The Icahn Genomics Institute, Icahn School of Medicine at Mount Sinai, New York, New York, USA

**Keywords:** influenza virus, mRNA export, NS1 (Non-Structural protein 1 of influenza A virus), NS1-BP (cellular NS1-binding protein), M1 (matrix protein 1 of influenza virus), NXF1 (nuclear RNA export factor-1), NXT1 (nuclear transport factor 2–like export factor 1), TREX-2 (transcription and export complex-2), GANP (germinal center–associated nuclear protein)

## Abstract

Influenza A viruses have eight genomic RNAs that are transcribed in the host cell nucleus. Two of the viral mRNAs undergo alternative splicing. The M1 mRNA encodes the matrix protein 1 (M1) and is also spliced into M2 mRNA, which encodes the proton channel matrix protein 2 (M2). Our previous studies have shown that the cellular Non-Structural protein 1 (NS1)-binding protein (NS1-BP) interacts with the viral NS1 and M1 mRNA to promote M1 to M2 splicing. Another pool of NS1 protein binds the mRNA export receptor nuclear RNA export factor-1 (NXF1), leading to nuclear retention of cellular mRNAs. Here, we show a series of biochemical and cell biological findings that suggest a model for nuclear export of M1 and M2 mRNAs despite the mRNA nuclear export inhibition imposed by the viral NS1 protein. NS1-BP competes with NS1 for NXF1 binding, allowing the recruitment of NXF1 to the M mRNAs after splicing. NXF1 then binds germinal center–associated nuclear protein, a member of the transcription and export complex-2. Although both NS1 and NS1-BP remain in complex with germinal center–associated nuclear protein-NXF1, they dissociate once this complex docks at the nuclear pore complex, and the M mRNAs are translocated to the cytoplasm. Since this mRNA nuclear export pathway is key for expression of M1 and M2 proteins that function in viral intracellular trafficking and budding, these viral–host interactions are critical for influenza virus replication.

Influenza A viruses (IAVs) cause high mortality rates, especially during pandemic years, therefore remain a major concern for public health (1, 2). The virus enters the host cell by endocytosis and induces fusion of the endosomal membrane with the viral membrane. This releases the eight unique viral genomic RNA (vRNA) segments into the host cell cytoplasm. These vRNAs are imported into the host cell nucleus *via* the nuclear pore complex (NPC). Once in the nucleus, the vRNAs are transcribed into mRNAs, two of which undergo alternative splicing. One of these is the M1 mRNA, which is translated into the M1 protein and can also be spliced into the M2 mRNA. The M1 protein has a role in viral intracellular trafficking, viral assembly, and as a structural component of the virion (3) while the M2 mRNA encodes the M2 ion channel that functions in viral entry and budding (3). Thus, both M1 and M2 proteins are critical for viral gene expression and replication. The viral NS1 mRNA encodes the Non-Structural protein 1 (NS1) and is also spliced into the mRNA that encodes the nuclear export protein or Non-Structural protein 2 (NS2). The NS1 protein is a virulence factor that inhibits interferon induction and response, regulates apoptosis through the PI3K pathway, and inhibits host gene expression (4, 5, 6). The latter includes inhibition of pre-mRNA splicing (7, 8), 3′ end processing (9, 10), and nuclear export of cellular mRNAs (11, 12). Opposite to the effects observed with cellular mRNAs, NS1 promotes viral M1 mRNA to M2 mRNA splicing (13, 14, 15) and nuclear export (13, 15, 16). Additionally, NS1 stimulates viral RNA synthesis and translation (4). NS2 is involved in the switch between viral RNA transcription and replication, and mediates nuclear export of vRNPs (17). Therefore both NS1 and NS2 are important for viral replication (3).

NS1 protein mediates splicing of influenza virus M1 mRNAs by interacting with several cellular factors in the nucleus. NS1 binds the cellular protein NS1-BP, which interacts with hnRNP K and influenza virus M1 mRNA and traffics the complex to nuclear speckles (13, 14, 15, 18). Nuclear speckles concentrates proteins involved in RNA processing and splicing, as well as mRNA export factors and regulators of these processes (19, 20). Our previous results suggest a model in which NS1-BP binds to the M1 mRNA pY-tract downstream the M2 mRNA 5′ splice site to inhibit premature splicing while hnRNP K binds to a pC-tract on M1 mRNA. At nuclear speckles, NS1 dissociates and hnRNP K recruits U1 small nuclear ribonucleoprotein to start splicing of M1 to M2 mRNA. While the interaction between NS1-BP and viral M1 mRNA decreases upon mutation of the pY-tract, NS1-BP can still interact with NS1 and hnRNP K. Low levels of NS1-BP or infection with influenza virus that lacks NS1 prevents proper M1 mRNA trafficking to nuclear speckles (13), which inhibits splicing of M1 to M2 mRNA (13, 14, 15, 18). NS1-BP and hnRNP K also regulate splicing of a subset of cellular mRNAs, which have pY- and pC-tracts downstream of their 5′ splice site.

After splicing, nuclear export of cellular mRNAs is mediated by the intranuclear protein transcription and export complex (TREX) that links transcription, mRNA processing, and nuclear export. TREX consists of a hexameric core termed THO (THOC1–3, 5–7), the RNA helicase UAP56 (56 kDa U2AF-associated protein; DDX39B), the adaptor protein ALYREF (THO Complex subunit 4), and additional proteins (21, 22). During gene expression, the TREX complex is recruited co-transcriptionally and pre-mRNA processing then mediates TREX assembly on the transcript (21). Through the combined action of TREX complex and other mRNA export factors, the major mRNA export receptor nuclear RNA export factor-1 (NXF1)-nuclear transport factor 2–related export protein (NXT1) heterodimer is recruited to the mRNA to mediate nuclear export through the NPC (21). At the nucleoplasmic side of the NPC, NXF1 can associate with another complex termed TREX-2 (consists of germinal center–associated nuclear protein [GANP]; PCI domain containing 2; deleted in spilt hand/spilt foot; enhancer of yellow 2 homolog; and Centrins) through its interaction with GANP (23). We and others have shown that the human TREX-2 component GANP mediates nuclear export of a subset of cellular mRNAs and of influenza virus mRNAs from diverse strains, including A/WSN/33 (H1N1), A/California/04/2009 (H1N1), A/Wyoming/3/2003 (H3N2), and A/Vietnam/1203/2004 (H5N1 HALO) (24, 25). During mRNA export, TREX-2 interacts with the nucleoporin TPR *via* GANP at the nucleoplasmic side of the NPC (25, 26). GANP binds the NXF1-NXT1 heterodimer (23), which then mediates translocation of the mRNA through the NPC.

During influenza infection, the viral NS1 protein interacts with the cellular mRNA export receptor NXF1-NXT1 resulting in inhibition of nuclear export of cellular mRNAs, a process that prevents proper expression of mRNAs that encode antiviral factors and make the translation machinery more available for viral mRNAs (11, 12, 27) (Fig. 1). In contrast, NS1 promotes M and HA mRNA nuclear export (13, 16) (Fig. 1). Additionally, the NS1 interacting partner NS1-BP also promotes M mRNA nuclear export and NS1-BP is not required for export of bulk cellular mRNAs but for a subset of mRNAs (13, 28) (Fig. 1). However, it is still unknown how NS1 and NS1-BP facilitate viral mRNA export (Fig. 1). Here, we uncovered a mechanism by which influenza virus M mRNAs are exported from the nucleus during infection. We identified the viral–host interactions involved in this nuclear export pathway that are critical for efficient expression of influenza virus M1 and M2 mRNAs. As these mRNAs encode proteins that are key players in viral intracellular trafficking and budding, this export pathway has an important role in influenza virus replication.

**Figure 1:**
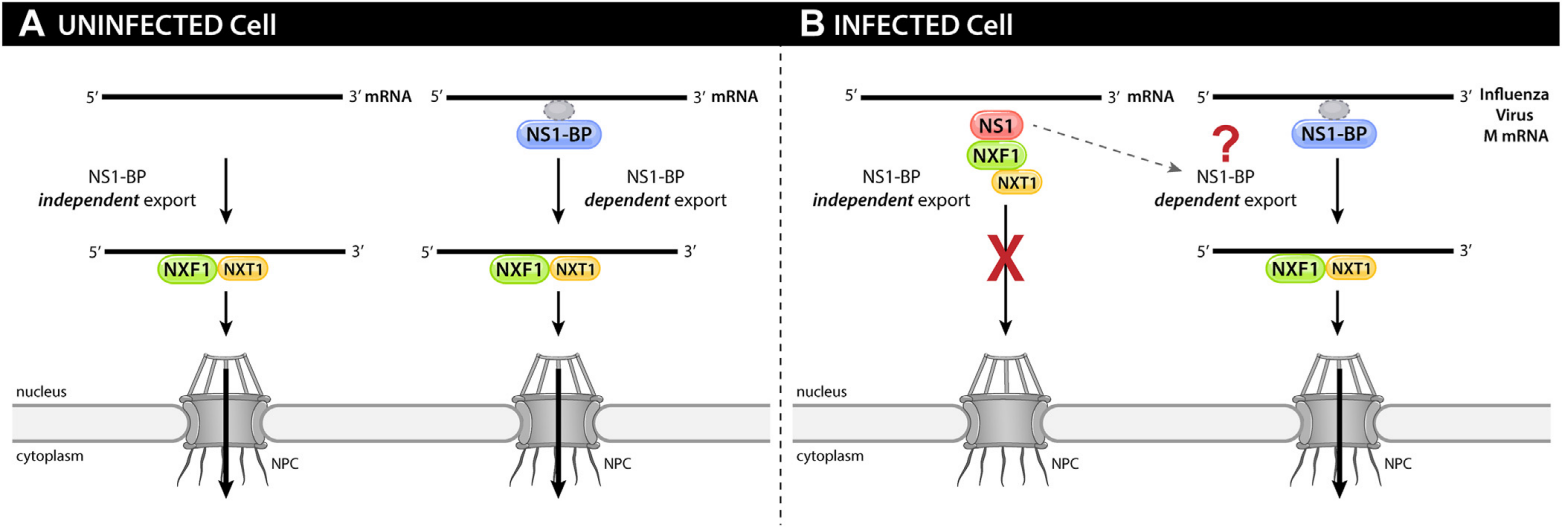
**Schematic representation of mRNA export pathways.***A*, in uninfected cells the cellular NS1-BP is involved in nuclear export of a subset of mRNAs. NXF1-NXT1 heterodimer is the main export receptor of mRNAs *via* the nuclear pore complex (NPC). *B*, in cells infected with influenza virus the viral protein NS1 inhibits cellular mRNA nuclear export by interacting with NXF1-NXT1 and preventing its docking at the NPC. However, NS1 and NS1-BP promote nuclear export of influenza virus mRNA. The mechanism involved in this process is the question addressed here. GANP, germinal center–associated nuclear protein; NS1, Non-Structural protein 1; NS1-BP, NS1-binding protein; NXF1, nuclear RNA export factor-1; NXT1, nuclear transport factor 2–related export protein.

## Results

### NS1-BP preferentially promotes nuclear export of influenza virus M mRNAs and interacts with the mRNA export receptor NXF1

**Figure 2:**
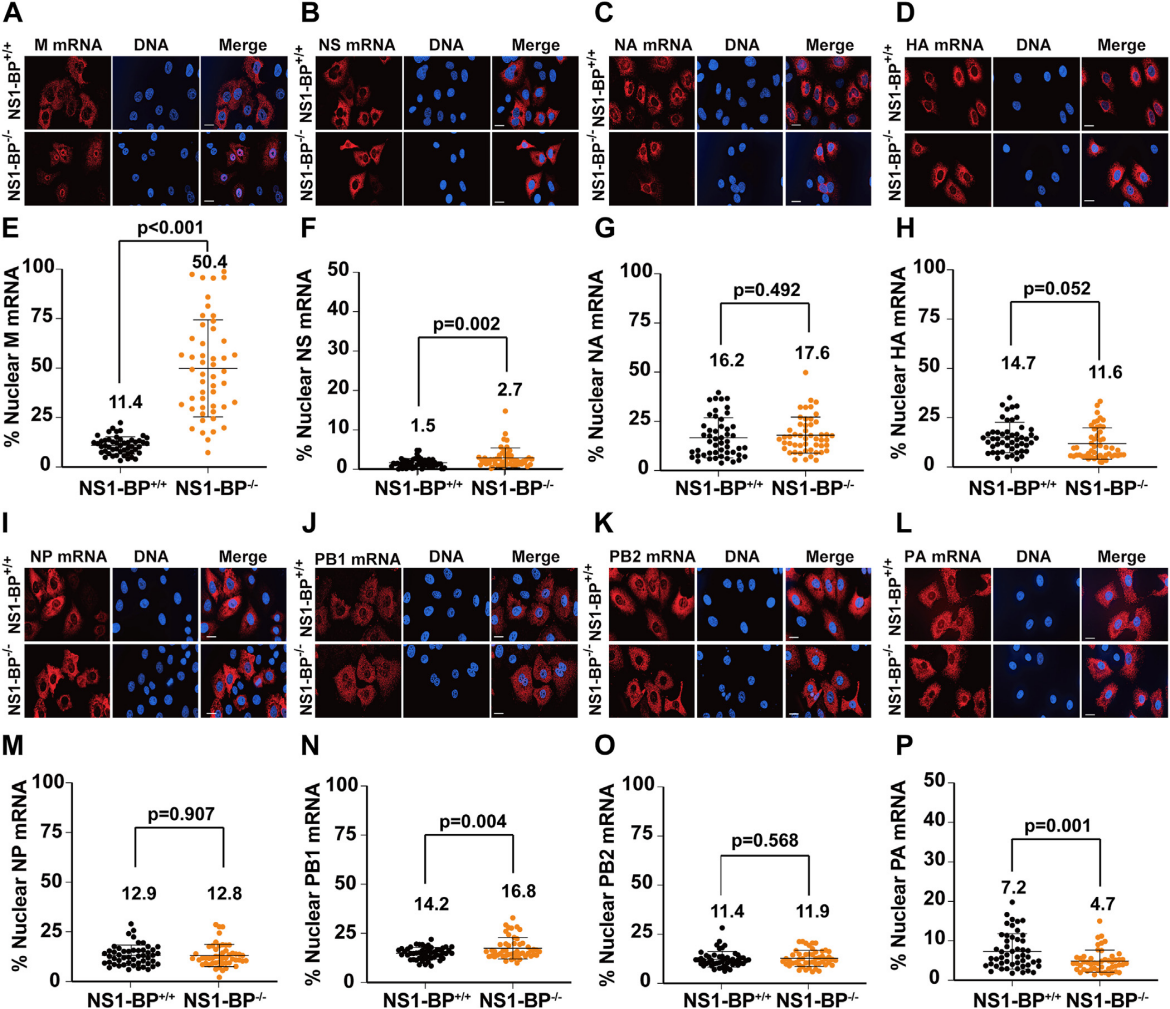
**NS1-BP preferentially promotes nuclear export of influenza virus M mRNAs.***A*-*D*, NS1-BP^+/+^ or NS1-BP^−/−^ cells were infected with influenza A virus (A/WSN/33) at MOI 2. After 8 h of infection, cells were fixed and subjected to smRNA FISH to detect viral M, NS, NA, and HA mRNAs. DNA was stained with Hoechst. The scale bar represents 5 μm. Data are representative of three independent experiments. *E-H*, fluorescence intensity of each viral mRNA was quantified in the whole cell and in the nucleus using Imaris software (Bit-plane). Percentage of nuclear values for each viral mRNA are shown for each cell (*dot*), *n =* 50 cells. Cells used for quantification were derived from three independent experiments. *I-L*, NS1-BP^+/+^ or NS1-BP^−/−^ cells were infected with influenza A virus (A/WSN/33) at MOI 2. After 8 h of infection, cells were fixed and subjected to smRNA FISH to detect viral NP, PB1, PB2, and PA mRNAs. DNA was stained with Hoechst. The scale bar represents 5 μm. Data are representative of three independent experiments. *M-P*, fluorescence intensity of each viral mRNA was quantified in the whole cell and in the nucleus using Imaris software (Bit-plane). Percentage of nuclear values for each viral mRNA are shown for each cell (*dot*), *n =* 50 cells. Cells used for quantification were derived from three independent experiments. Graphs show mean ± SD. *p* values were calculated using unpaired two tailed Student’s *t* test. *p* values < 0.05 are considered significant. MOI, multiplicity of infection; NS1, Non-Structural protein 1; NS1-BP, NS1-binding protein; smRNA FISH, single-molecule RNA FISH.

As mentioned above, M mRNAs include the M1 mRNA, which encodes the M1 protein, and the alternatively spliced M2 mRNA that encodes the M2 protein. Additionally, M1 mRNA can also be alternatively spliced into the so-called mRNA_3_ and, in certain strains such as A/WSN/33 virus, M1 mRNA is also spliced into mRNA4. It is still unknown whether mRNA_3_ encodes a functional peptide (29) and mRNA4 was shown to express an isoform of the M2 ion channel that is not present in all strains (30). Our previous findings demonstrated that NS1-BP and the viral NS1 protein promote M1 to M2 splicing and nuclear export, contributing to proper viral replication (13, 14, 15, 18, 28, 31). Here, we tested whether NS1-BP is required for nuclear export of other influenza virus mRNAs. We infected NS1-BP^+/+^ or NS1-BP^−/−^ cells with influenza A/WSN/33 virus and performed single-molecule RNA FISH (smRNA FISH), followed by quantification of fluorescence intensity in the nucleus and in the cytoplasm to determine the intracellular distribution of specific influenza virus mRNAs. As shown in Fig. 2*A*-P, while the intracellular localization of most influenza virus mRNAs is not affected by the absence of NS1-BP, nuclear export of influenza virus M mRNAs is significantly inhibited, as shown by both smRNA FISH and corroborated by nucleo-cytoplasmic fractionation followed by quantitative polymerase chain reaction (qPCR) (Figs. 2, *A* and *E* and S1).

**Figure 3:**
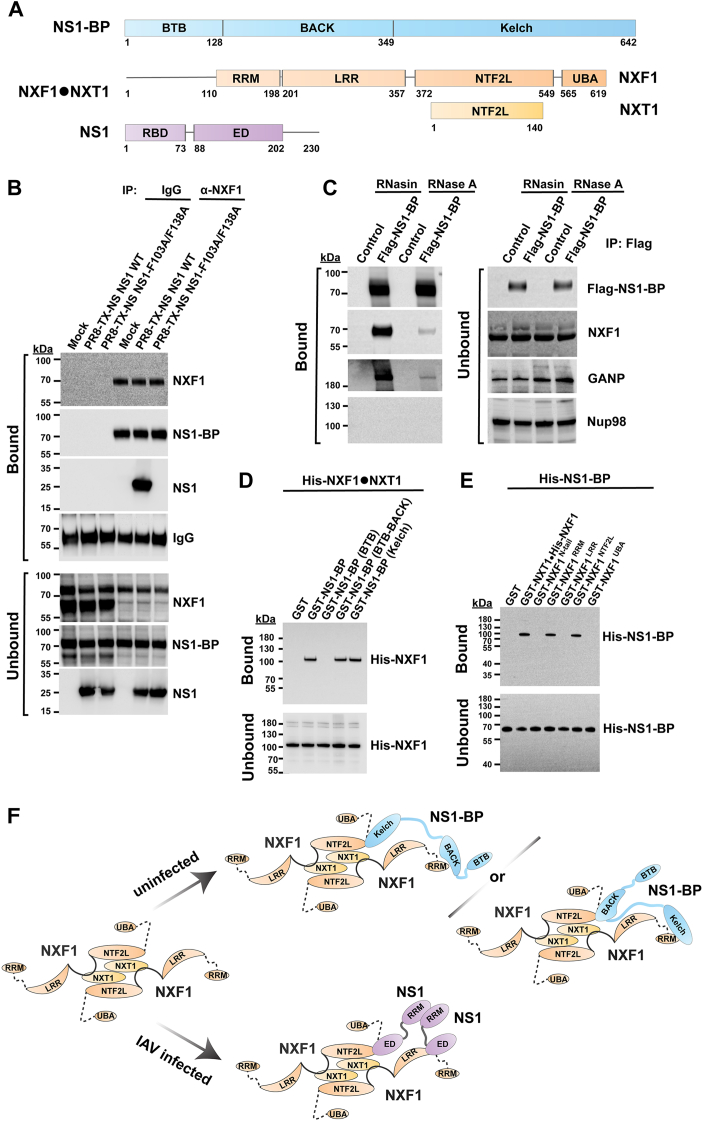
**NS1-BP directly interacts with the cellular mRNA export receptor NXF1.***A*, schematic representation of NS1-BP, NXF1-NXT1, and NS1 proteins and their domains. *B*, cell lysates from A549 cells infected with influenza A virus expressing WT NS1 protein (PR8-TX-NS NS1^WT^) or a mutant of NS1 (PR8-TX NS1 NS1^F103A/F138A^) were subjected to immunoprecipitation with anti-NXF1 antibody, followed by Western blots with specific antibodies against the depicted proteins. *C*, cell extracts from A549 cells stably expressing NS1-BP WT protein were subjected to immunoprecipitation with anti-Flag antibody in the presence of RNasin or RNase A, and immunoprecipitated proteins were immunoblotted with specific antibodies against the depicted proteins. *D*, *in vitro* binding assay performed with purified His-NXF1-NXT1 proteins and GST-NS1-BP, GST-NS1-BP (BTB), GST-NS1-BP (BTB-BACK), GST-NS1-BP (Kelch), or control GST immobilized on beads. NXF1 was detected by Western blot using a NXF1 antibody. *E*, *in vitro* binding assay performed with purified His-NS1-BP protein and GST-NXT1•HisNXF1, GST-NXF1^N-tail^, GST-NXF1^RRM^, GST-NXF1^LRR^, GST-NXF1^NTF2L^, GST-NXF1^UBA^, or control GST immobilized on beads. NS1-BP was detected by Western blot using a NS1-BP antibody. Bound and unbound fractions are shown. *F*, schematic representation of NXF1-NXT1, NS1, and NS1–BP interactions and their respective domains. Based on the crystal structure, NXF1-NXT1 dimer interacts with the influenza virus NS1 homodimer. NS1 interacts with the LRR and NTF2L domains of NXF1, preventing NXF1 docking at nucleoporins (Nups) (12). NS1-BP also interacts with the NTF2L domain of NXF1 in addition to the RRM domain, and two possible modes of interaction are depicted. GST, glutathione-S-transferase; LRR, leucine-rich repeat; NS1, Non-Structural protein 1; NS1-BP, NS1-binding protein; NTF2L, nuclear transport factor 2-like domain; NXF1, nuclear RNA export factor-1; NXT1, nuclear transport factor 2–related export protein.

We next sought to determine the mechanism by which NS1-BP mediates this nuclear export function. NS1-BP functions as a dimer and has a BTB domain (dimerization function), followed by the BACK and Kelch domains (Fig. 3*A*) that are involved in interactions with splicing and mRNA export factors, among which is the viral NS1 protein (Fig. 3*A*) (15) that promotes nuclear export of influenza virus M mRNAs (13, 16). However, NS1 interacts with the mRNA export receptor NXF1 to inhibit nuclear export of cellular mRNAs (11, 15). We have tested the interaction between NS1-BP and key members of the mRNA nuclear export machinery and found that NS1-BP interacts with the mRNA export receptor NXF1 in the presence or absence of infection. As shown in Fig. 3*B*, A549 cells were infected with influenza virus expressing WT NS1 protein or a mutant of NS1 (F103A/F138A) that does not interact with NXF1, as we previously reported (12). Cell extracts from mock or infected cells were then subjected to immunoprecipitation with control IgG or anti-NXF1 antibodies. We show that NS1-BP interacts with NXF1 in infected cells independently of the known interactions between the viral NS1 protein and NS1-BP (15, 18, 32) or NS1 and NXF1 (11, 12), as the interaction between NS1-BP and NXF1 is detected in mock-infected cells (Fig. 3*B*). This is further corroborated in cells stably expressing 3XFlag-NS1-BP from which NS1-BP was immunoprecipitated with anti-Flag antibody and compared to cells expressing the 3XFlag epitope alone. Again, we found that NS1-BP also binds NXF1 in the absence of infection (Fig. 3*C*). Since these cell extracts were incubated in the presence of RNasin or RNase A, we observed that the interaction between NS1-BP and NXF1 is partially dependent on RNA (Fig. 3*C*). In contrast, NS1-BP did not interact with the nucleoporin Nup98, which serves as negative control (Fig. 3*C*).

Next, we tested whether NS1-BP directly interacts with NXF1 using purified proteins in *in vitro* binding assays. We generated and purified glutathione-*S*-transferase (GST)-tagged full-length NS1-BP or GST-tagged NS1-BP domains (BTB, BTB-BACK, or Kelch) and incubated these individual proteins with purified His-NXF1-NXT1. GST pull down was performed and showed that NXF1-NXT1 binds to the BACK and Kelch domains of NS1-BP (Fig. 3*D*). To then map the domains of NXF1 involved in NS1–BP interaction, we purified GST-tagged NXT1 in complex with His-NXF1 full length or with NXF1 domains: N-tail flexible region (amino acids 1–109), the RNA-binding domains [RNA recognition motif (RRM) and leucine-rich repeat], the nuclear transport factor 2-like domain (NTF2L) that binds both mRNA and nucleoporins, and an ubiquitin-associated domain], which is also a nucleoporin-binding domain (Fig. 3*A*). The NTF2L domain forms a heterodimer with NXT1/p15, which is a 15-kDa protein that also has an NTF2-like fold (Fig. 3*A*). We found that NS1-BP interacts with the RRM and the nucleoporin-binding site (NTF2L) (Fig. 3*E*). The latter suggests that NS1-BP should be dissociated from NXF1 before NXF1 docks at nucleoporins in the NPC. This is corroborated by the lack of interaction between NS1-BP and Nup98 observed in Fig. 3*C* and also by the absence of nucleoporins in the proteome of NS1-BP interacting partners as we previously reported (15). Fig. 3*F* summarizes these interactions described above. Taken together, these findings suggest that NS1–BP interaction with NXF1 may occur prior to NXF1 docking to the NPC.

### NXF1 interactions with NS1-BP or with NS1 are localized mostly in the nucleus

**Figure 4:**
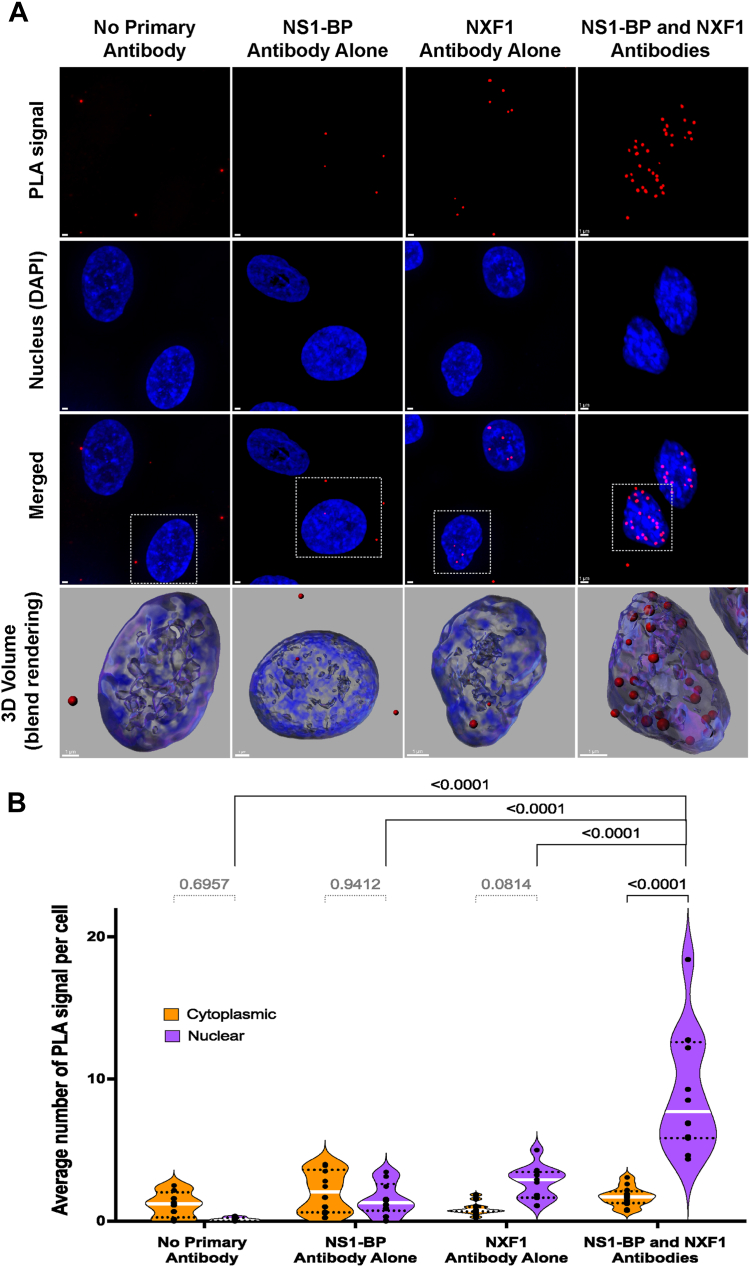
**NS1-BP and NXF1 interaction occurs in the nucleus.***A*, A549 cells were subjected to proximity ligation assay (PLA) to detect the interaction between NS1-BP and NXF1 proteins *in situ*. The interaction by PLA is detected by fluorescent probes (*red dots*; λem = 624 nm, TRITC filter). Hoechst staining labels the nuclei (*blue*). The scale bar represents 1 μm. See Fig. S2 for the entire field of view. The *bottom row panels* are 3D projections of chromatin merged surface with the PLA signals detecting the protein complexes in the selected cells (*dashed line squares*). *B*, quantification of average number of PLA signals per cell were obtained from 88 (no primary antibody), 111 (NS1-BP primary antibody), 99 (NXF1 primary antibody), and 154 (NS1-BP1 and NXF1 antibodies) cells. *Dots* show the numbers of PLA signals in cells per microscope field of view. The *dashed lines* represent quartiles, and the *white line* represents median value. Data are representative of three independent experiments. Statistical analysis was performed using one-way ANOVA with a Tukey post test, and *p* values are depicted in the figure. NXF1, nuclear RNA export factor-1; NXT1, nuclear transport factor 2–related export protein; NS1, Non-Structural protein 1; NS1-BP, NS1-binding protein; TRITC, tetramethylrhodamine.

**Figure 5:**
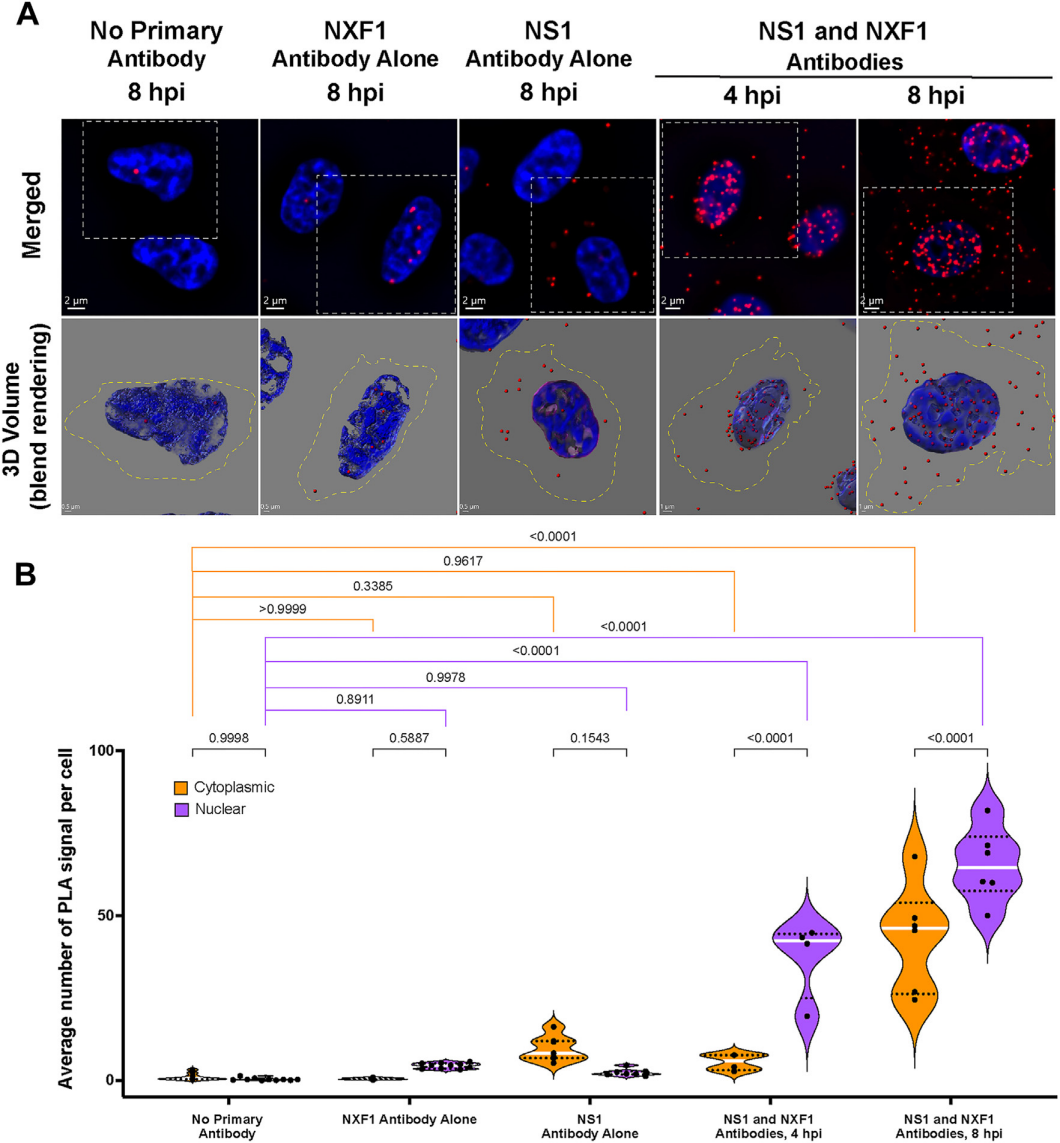
**Temporospatial dynamics of NS1 and NXF1 interaction during infection.***A*, A549 cells infected with influenza virus were subjected to PLA to detect the interaction between NS1 and NXF1 proteins *in situ* at 4 hpi (hours post infection) or 8 hpi. The interaction by PLA is detected by fluorescent probes (*red dots*; λem = 624 nm, TRITC filter). Hoechst staining labels the nuclei (*blue*). The scale bar represents 2 μm. See Fig. S3 for the entire field of view. The *bottom row panels* are 3D projections of chromatin merged surface with the PLA signals detecting the NS1–NXF1 complexes in the selected cells (*dashed line squares*), the scale bar represents 0.5 μm. Cell boundaries are marked by *yellow dashed lines*. *B*, quantification of average number of PLA signals per cell were obtained from 85 (no primary antibody, 8 hpi), 95 (NS1 primary antibody, 8 hpi), 71 (NXF1 primary antibody, 8 hpi), 69 (NS1 and NXF1 antibodies, 4 hpi), and 77 (NS1 and NXF1 antibodies, 8 hpi) cells. The *dashed lines* represent quartiles, and the *white line* represents median value. Data are representative of three independent experiments. Statistical analysis was performed using one-way ANOVA with a Tukey post test, and *p* values are depicted in the figure. NS1, Non-Structural protein 1; NS1-BP, NS1-binding protein; PLA, proximity ligation assay; NXF1, nuclear RNA export factor-1; NXT1, nuclear transport factor 2–related export protein; TRITC, tetramethylrhodamine.

**Figure 6:**
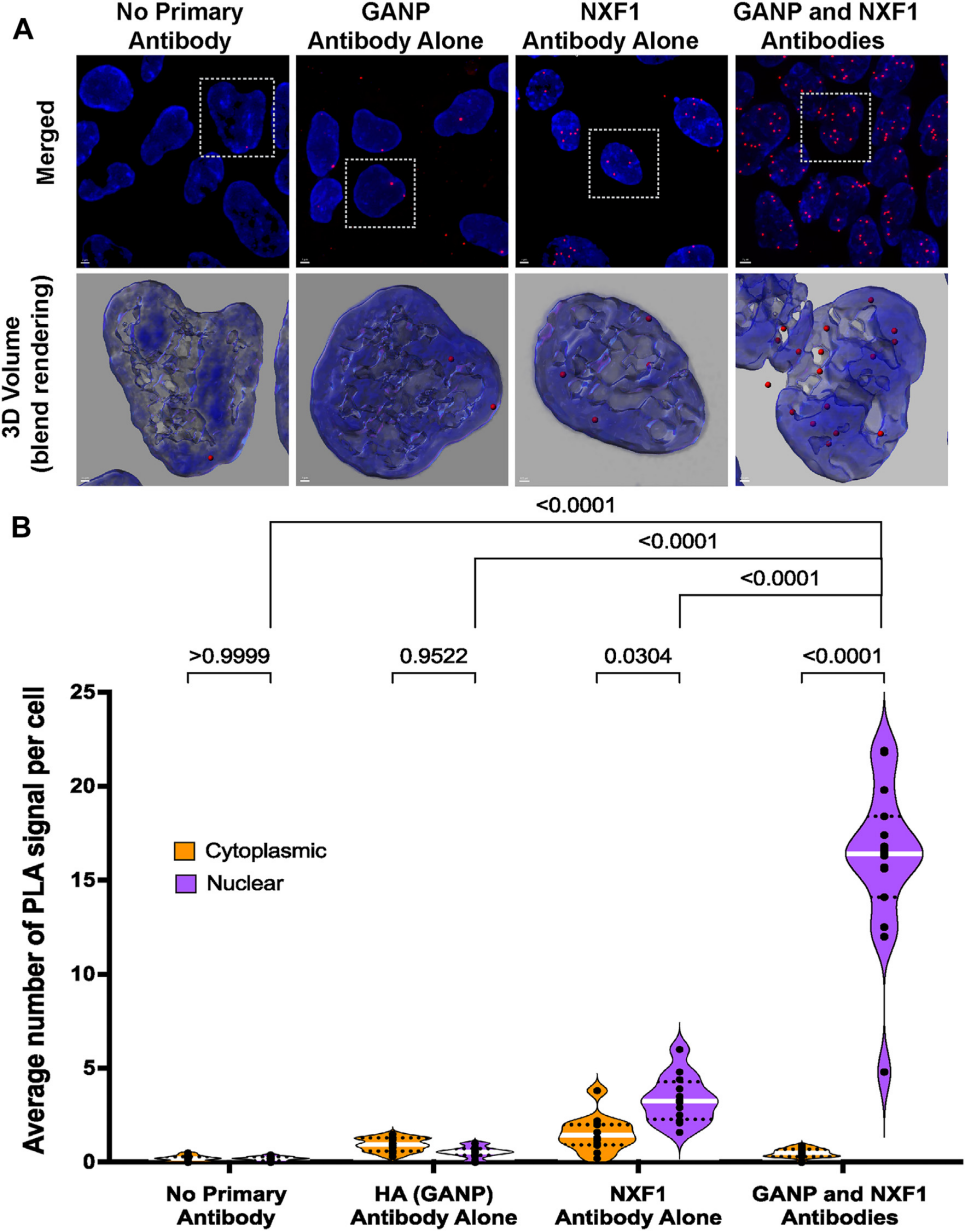
**GANP and NXF1 interaction is detected in the nucleoplasm and in the nuclear periphery.***A*, DLD1-^HA-AID^GANP cells were subjected to PLA to detect the interaction between GANP and NXF1 proteins *in situ*. The interaction by PLA is detected by fluorescent probes (*red dots*; λem = 624 nm, TRITC filter). Hoechst staining labels the nuclei (*blue*). The scale bar represents 1 μm. The *bottom row panels* are 3D projections of chromatin merged surface with the PLA signals detecting the GANP–NXF1 complexes in the selected cells (*dashed line squares*), the scale bar represents 0.5 μm. See Fig. S4 for the entire field of view. *B*, quantification of average number of PLA signals per cell were obtained from 168 (no primary antibody), 104 (GANP (anti-HA) primary antibody), 113 (NXF1 primary antibody), and 177 [GANP (anti-HA) and NXF1 antibodies] cells. *Dots* show the numbers of PLA signals in cells per microscope field of view. The *dashed lines* represent quartiles, and the *white line* represents median value. Data are representative of three independent experiments. Statistical analysis was performed using one-way ANOVA with a Tukey post test, and *p* values are depicted in the figure. GANP, germinal center–associated nuclear protein; NS1, Non-Structural protein 1; NS1-BP, NS1-binding protein; NXF1, nuclear RNA export factor-1; NXT1, nuclear transport factor 2–related export protein; PLA, proximity ligation assay; TRITC, tetramethylrhodamine.

We next performed proximity ligation assays (PLAs) to determine the intracellular localization of the interaction between NS1-BP and NXF1 in intact cells. To this end, antibodies that recognize NS1-BP and NXF1 interact with secondary antibodies linked to oligos, which prime DNA amplification that is then hybridized to fluorescent oligos. The PLA signal detecting the interaction between NS1-BP and NXF1 is revealed by red dots (Fig. 4*A*), which were quantified in the nucleus and in the cytoplasm (Fig. 4*B*). In the absence of primary antibodies or in the presence of only one of the primary antibodies, background signal is detected. However, when both NS1-BP and NXF1 antibodies are added there is a robust increase in intranuclear signal, indicating the interaction between both proteins. The NS1–BP interaction with NXF1 can also be observed in 3D (Fig. 4*A*), showing a prevalent localization inside the nucleus and only a few interactions are detected in the nuclear periphery. We have also followed the NXF1–NS1 interaction inside the nucleus by PLA over time of infection (Fig. 5). This complex is localized in the nuclear interior and at the nuclear periphery at 4 h postinfection (Fig. 5, *A* and *B*), suggesting a potential function for the viral NS1 protein in promoting or recruiting NXF1 to the viral mRNP. At 8 h postinfection, we observed NXF1–NS1 interaction in the nucleus and in the cytoplasm (Fig. 5, *A* and *B*). These results indicate that NS1 may prevent nuclear import of NXF1 in addition to disrupting and/or repurposing NXF1 functions in the nucleus. We have also corroborated the GANP–NXF1 interaction by PLA. We detected this interaction both inside the nucleus and at the nuclear periphery (Fig. 6, *A* and *B*). Thus, these interactions described above have been detected both biochemically and *in situ*.

### NS1-BP competes with the viral NS1 protein for binding to NXF1-NXT1

**Figure 7:**
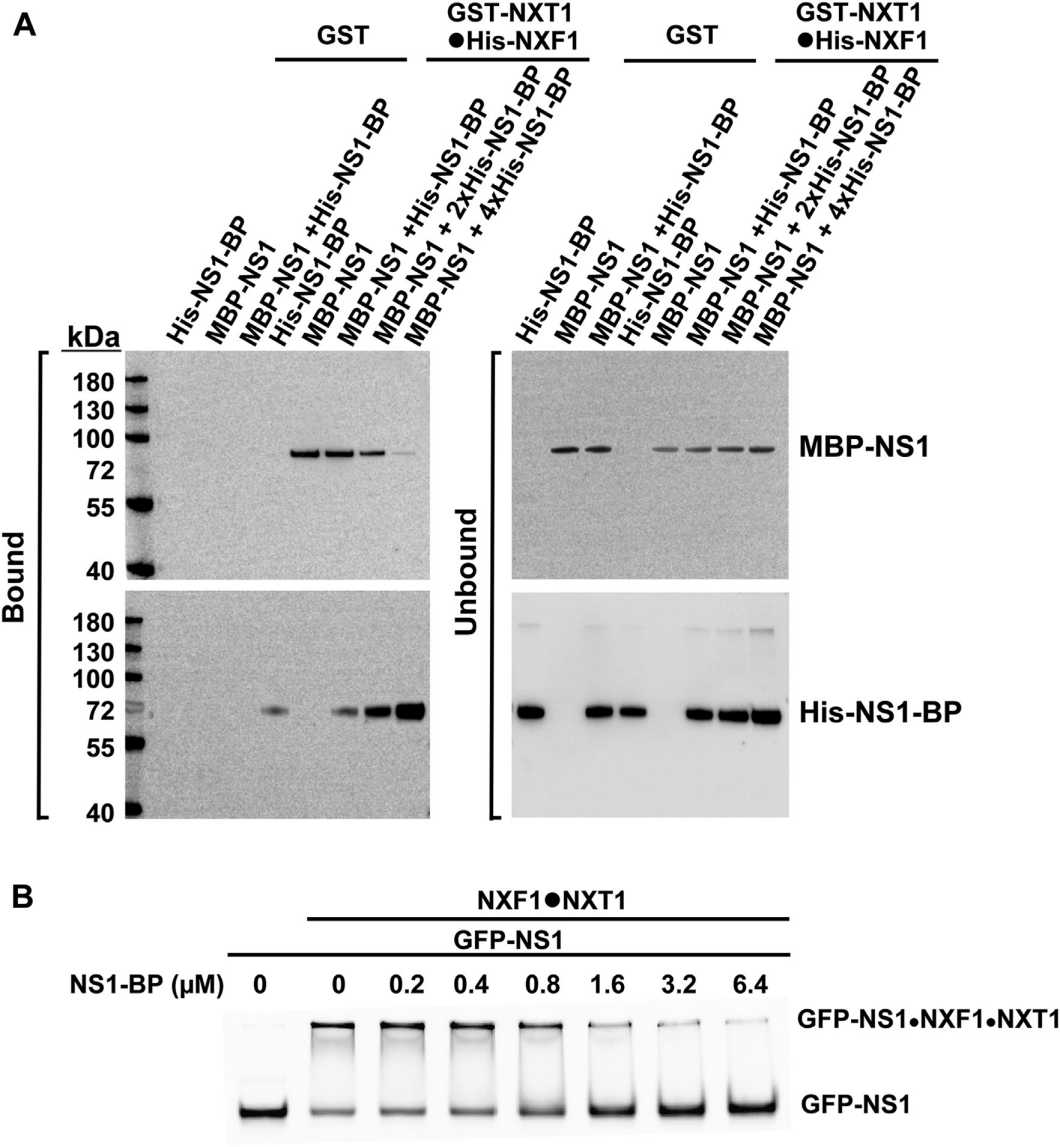
**Cellular NS1-BP protein competes with viral NS1 protein for NXF1 binding.***A*, GST-NXT1•HisNXF1 bound to MBP-NS1 was immobilized on GSH beads and MBP-NS1 was then dissociated by 2x and 4x molar excess of His-NS1-BP protein. NS1 and NS1-BP proteins were detected by Western blot using NS1 and NS1-BP antibodies. *B*, NS1-BP competes with NS1 for binding to NXF1-NXT1 in a concentration-dependent manner. EMSA was carried out with GFP-NS1 at 0.1 μM, NXF1-NXT1 at 3 μM, and NS1BP at the indicated concentrations. The gel was visualized by the fluorescence from GFP-NS1. EMSA, Electrophilic mobility shift assay; GST, glutathione-S-transferase; NS1, Non-Structural protein 1; NS1-BP, NS1-binding protein; NXF1, nuclear RNA export factor-1; NXT1, nuclear transport factor 2–related export protein.

We have previously shown that the viral NS1 protein binds the nucleoporin-binding domain of NXF1 (NTF2L) (12) and NS1-BP interacts with both the NTF2L and RRM domains of NXF1 (Fig. 2). We therefore asked whether NS1-BP and NS1 compete for NXF1 binding at the NTF2L domain. We performed GST pull-down assays with purified GST alone or GST-NXT1•His-NXF1 incubated with increasing amounts of NS1-BP (Fig. 7*A*). We show that increasing amounts of NS1-BP displace NS1 binding to NXF1-NXT1 (Fig. 7*A*). We have also examined the effect of NS1-BP on the NS1 interaction with NXF1-NXT1 using an electrophilic mobility shift assay with purified recombinant proteins. NS1 was tagged with GFP to visualize the protein. NXF1-NXT1 shifts the NS1 band to lower mobility, indicating the formation of the NS1–NXF1–NXT1 complex (Fig. 7*B*). As the concentration of NS1-BP increases per reaction, GFP-NS1 progressively dissociates from its complex with NXF1-NXT1. Our results indicate that NS1-BP competes with NS1 for binding to NXF1-NXT1, although there is the possibility of a transient tetrameric complex NS1•NS1-BP•NXF1•NXT1 as NS1-BP can bind to the RRM domain of NXF1, while NS1 could bind the NTF2L domain.

**Figure 8:**
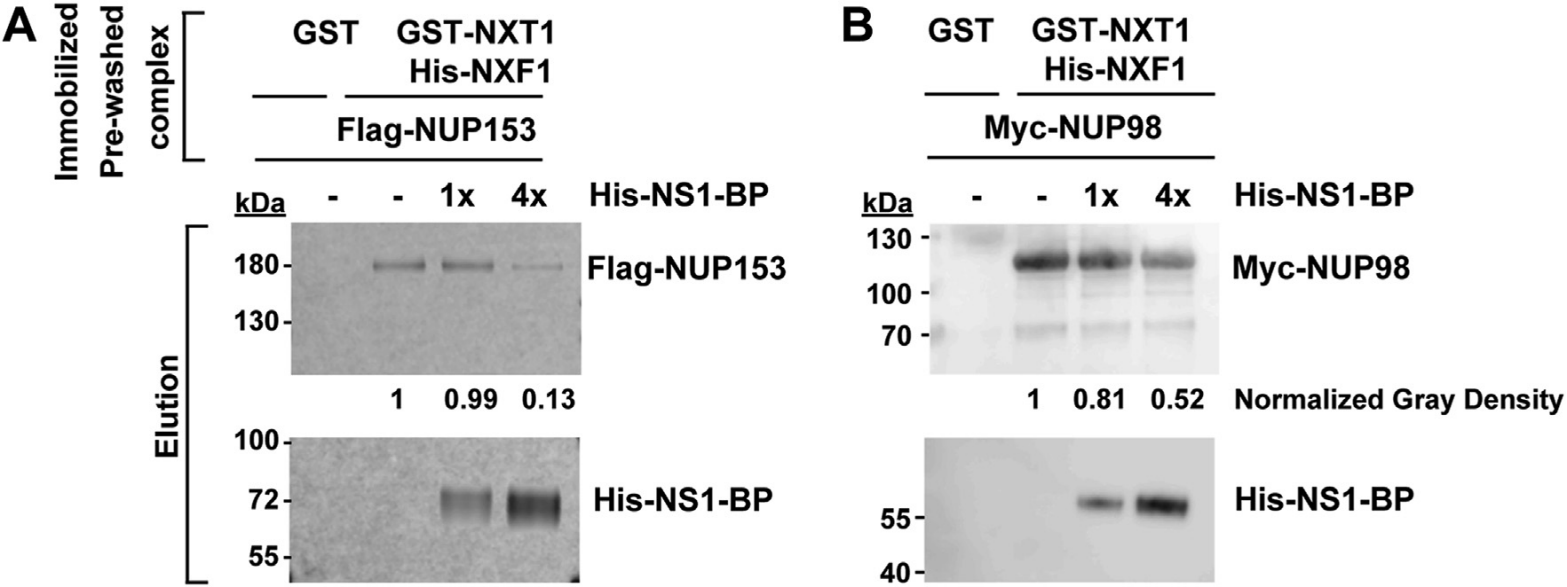
**NS1-BP competes with NUP153 and NUP98 for the nucleoporin-binding site on NXF1.** Purified recombinant GST-NXT1•His–NXF1complex bound to (*A*) Flag-NUP153 or (*B*) Myc-NUP98 was immobilized on GSH beads and then (*A*) Flag-NUP153 or (*B*) Myc-NUP98 was dissociated from the complex by 4x molar excess His-NS1-BP protein addition. Flag-NUP153 and Myc-NUP98 were detected by Western blot using anti-Flag antibody and NUP98 antibody, respectively. GST, glutathione-S-transferase; NS1, Non-Structural protein 1; NS1-BP, NS1-binding protein; NXF1, nuclear RNA export factor-1; NXT1, nuclear transport factor 2–related export protein.

Since NS1-BP interacts with the nucleoporin-binding domain of NXF1 (NTF2L), we reasoned that NS1-BP would likely compete with nucleoporins for NXF1 binding. To this end, we used NUP153 and NUP98 as model Phenylalanine - Glycine-containing nucleoporins previously shown to interact with NXF1 (33). We performed in *vitro* binding assays with Flag-NUP153 (Fig. 8*A*) or Myc-NUP98 (Fig. 8*B*) expressed in the rabbit reticulocyte lysate, which were incubated with purified His-NXF1•GST-NXT1, washed, and then incubated in the presence of increasing amounts of NS1-BP. As predicted based on the results above, NS1-BP competed with Nup153 and Nup98 for the nucleoporin-binding site on NXF1. These results again suggest that NS1-BP likely dissociates from NXF1 prior to NXF1 docking at the NPC. Taken together, the viral NS1 protein and the cellular NS1-BP protein likely handover the influenza virus M mRNAs to the mRNA nuclear export machinery [NXF1-GANP (TREX2)].

## Discussion

We have previously shown that bulk cellular mRNA nuclear export is not altered by NS1-BP knockout or knockdown, but a subset of cellular mRNAs and influenza virus M mRNAs require NS1-BP for proper nuclear export (13, 15, 28). Among the selected cellular mRNAs analyzed are mRNAs that encode proteins involved in immunity and metastatic cancer (15). Regarding influenza virus, while the viral NS1 protein inhibits nuclear export of a large number of cellular mRNAs (11, 12, 34), both viral NS1 protein and its cellular binding partner NS1-BP promote nuclear export of selected influenza virus mRNAs, as we and others have shown (13, 15, 16, 28). Thus, the studies reported here on the interplay between the cellular NS1-BP protein with the cellular mRNA nuclear export machinery uncover a nuclear export mechanism for an important subset of cellular mRNAs and for influenza virus M mRNAs.

**Figure 9:**
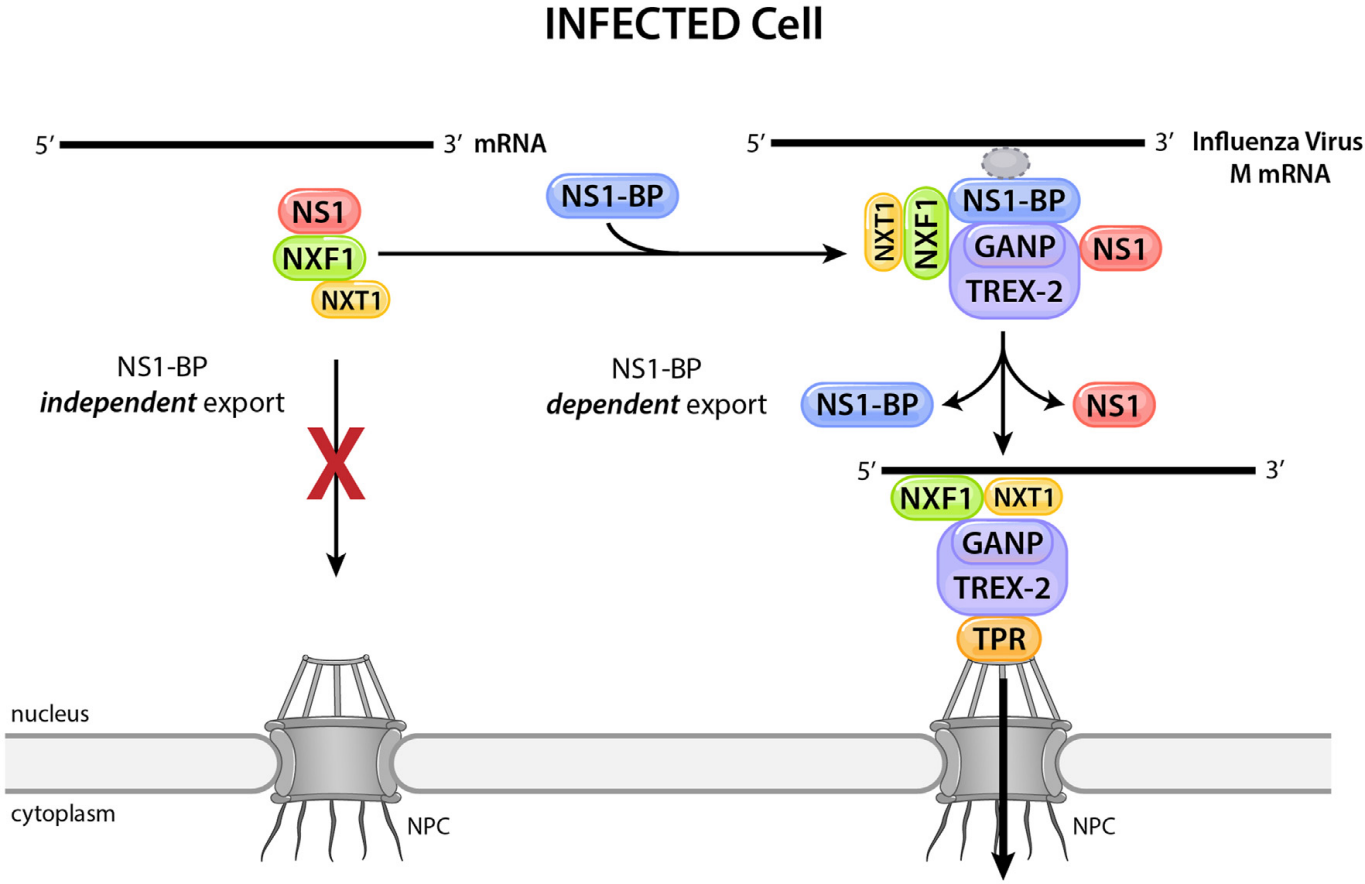
**Model for NS1-BP–mediated nuclear export.** During influenza virus infection, the viral NS1 protein interacts with the cellular mRNA export receptor NXF1-NXT1 and inhibits nuclear export of cellular mRNAs that are independent on the cellular NS1-BP protein for their nuclear export. Some of these mRNAs encode antiviral factors. To mediate nuclear export of influenza virus M mRNAs, the cellular NS1-BP protein competes with the viral NS1 protein for NXF1 binding. NXF1 interacts with GANP, a member of the TREX-2 complex, and both NS1-BP and NS1 transiently remain in the complex with GANP and NXF1. Once GANP (TREX-2) docks at the NPC, NS1-BP and NS1 are dissociated from GANP and the NXF1-bound M mRNAs are then translocated through the NPC to the cytoplasm. In the absence of infection, the pathway would have the same steps except for the presence of NS1 and inhibition of cellular mRNA nuclear export. GANP, germinal center–associated nuclear protein; NPC, nuclear pore complex; NS1, Non-Structural protein 1; NS1-BP, NS1-binding protein; NXF1, nuclear RNA export factor-1; NXT1, nuclear transport factor 2–related export protein; TREX, transcription and export complex.

We and others have previously shown that NS1-BP interacts with the influenza virus NS1 protein (13, 15, 32) and that they are critical for targeting the influenza virus M1 mRNA to nuclear speckles for splicing (13). Our studies suggested a model in which, at nuclear speckles, NS1-BP interacts with hnRNP K and NS1 dissociates from M1 mRNA for the recruitment of U1 small nuclear RNA by hnRNP K to mediate splicing of M1 to M2 mRNA (13, 14, 15, 18, 31). Here, we addressed the pathway by which these mRNAs are exported from the nucleus *via* NS1-BP. As illustrated in Fig. 9, the nucleoplasmic pool of the viral NS1 protein interacts with the cellular mRNA export receptor NXF1 to inhibit cellular mRNA nuclear export, including mRNAs that encode antiviral factors (11, 12). The cellular NS1-BP protein then competes with the viral NS1 protein for NXF1 binding and form a complex with GANP (TREX-2 complex), which we have shown to be required for nuclear export of influenza virus mRNAs (24). Once GANP-bound NXF1 docks at the NPC, NS1-BP and NS1 are dissociated from GANP. Next, NXF1-bound RNA is translocated through the NPC to the cytoplasm for translation. In the absence of infection, the pathway would have the same steps except for the presence of NS1 and would be involved in nuclear export of a subset of cellular mRNAs (Fig. 9). Thus, NS1-BP recruits a subset of mRNAs to the mRNA nuclear export machinery.

The viral NS1 protein is multifunctional as it inhibits cellular pathways in the nucleus and in the cytoplasm (4). We have previously shown the interaction between NS1 and NXF1 in cell extracts, at the structural level, and have also generated a recombinant virus in the interacting surface that demonstrated its function in inhibiting nuclear export of cellular mRNAs by preventing docking of NXF1 to the NPC (11, 12). In contrast, NS1 promotes viral mRNA nuclear export (13, 16). Here, our results suggest that the viral NS1 protein may be “stealing” NXF1 from the cellular machinery to hand it over to viral mRNAs for their nuclear export and thereby inhibiting nuclear export of cellular mRNAs from the NS1-BP–independent nuclear export pathway. In the case of influenza virus M1 and M2 mRNAs, NS1-BP bound to M1 and M2 mRNAs competes with NS1 for NXF1 binding. NXF1 then interacts with the TREX-2 complex *via* GANP and both NS1-BP and NS1 can still be associated with this complex. However, NS1-BP and NS1 are dissociated from the NXF1-GANP (TREX2) once it docks at the NPC (Fig. 9).

This model is consistent with our results showing the localization of NS1–BP interaction with NXF1 inside the nucleus (not at the nuclear periphery) by PLA. This is further corroborated by our previously reported proteomics studies in which we did not detect nucleoporin interaction with NS1-BP. Moreover, we showed that NS1-BP competes with nucleoporins for NXF1 binding. Therefore, NS1-BP likely dissociates from NXF1 and TREX-2 prior to NPC docking. Similarly, NS1 is likely not in complex with NXF1 during its interaction with the NPC as NS1 would block NXF1 docking at the NPC (12), resulting in inhibition viral mRNA nuclear export. Thus, NS1 probably promotes viral mRNA nuclear export prior to NXF1 interaction with the NPC. We have also detected a significant number of NS1–NXF1 interactions in the cytoplasm at 8 h postinfection. These data suggest that in addition to the nuclear NXF1–NS1 interaction, cytoplasmic NS1–NXF1 interaction further contributes to inhibition of cellular mRNA nuclear export by sequestering a pool of NXF1 in the cytoplasm. Viral M mRNA nuclear export is not affected at this time of infection as most viral mRNAs have already been exported into the cytoplasm. By further retaining cellular mRNAs in the nucleus when viral mRNAs are already in the cytoplasm, the cytoplasmic NS1–NXF1 interaction likely promotes translation of viral mRNAs by making the translation machinery more available to these mRNAs. The nuclear export inhibition of cellular mRNAs at this late time point may also prevent translation of mRNAs that encode antiviral factors, which further promote viral gene expression.

Both NS1 and NS1-BP mediate efficient nuclear export of viral M mRNAs but a small subset of M mRNAs can still be exported from the nucleus in the absence of both proteins, likely *via* other adaptor proteins (13, 16). Influenza virus lacking NS1 (35) or having a mutation in the NXF1-binding site (12) are attenuated, indicating that that NS1 promotes influenza virus replication at least in part by inhibiting expression of mRNAs that encode antiviral factors, as we reported (12). Additionally, low levels of NS1-BP inhibit virus replication (18). Thus, these results highlight the importance of NS1 and NS1-BP for efficient viral gene expression and replication and have implications for designing strategies to identify inhibitors that could potentially target NS1 or NS1–BP interactions with the TREX-2 pathway to inhibit virus replication. In sum, our findings reveal previously unknown interactions of the cellular protein NS1-BP with the mRNA nuclear export machinery and a mechanism involved in the functions of NS1-BP and the viral NS1 protein in promoting nuclear export of influenza virus M mRNAs. This is a key mechanism for influenza virus replication as M1 and M2 mRNAs encode proteins that are critical for viral intracellular trafficking and budding.

## Experimental procedures

### Plasmids

Full-length human NS1-BP (NM_006469.4; aa 1–642) and mutants encoding amino acids 1 to 234, 1 to 350, and 351 to 642 were cloned into BamH I and Xho I sites of the pGEX-6p-1 vector with the GST tag at the amino terminus (15). Full-length NS1-BP was cloned into the Nco I and Xho I sites of the pET-28b vector with the 6xHis tag at the carboxyl terminus (15). Full-length NS1-BP were cloned into the Xho I and Smal I sites of the pCI-3xFlag-Neo vector with the 3xFlag tag at the amino terminus (15). Constructs for NXF1 and NXT1 are described in Zhang *et al.* (12). Full-length IAV strain NS1 from A/WSN/1933 was cloned into Not I and BamH I sites of the pMALTEV vector (pMAL with tobacco etch virus sequence-specific cysteine protease cleavage site) with MBP fusion at the amino terminus (15). pMAX-myc-NUP98 was generated as previously described (36). Full-length human NUP153 (residues 1–1475) was cloned into the XhoI and Smal I sites of the pCI-neo-3 × Flag vector with the 3 × Flag tag at the N terminus to generate plasmids encoding Flag–Nup153 (12). NS1(R38A/K41A) was cloned with an N-terminal His tag and GFP tag into a pProEx HTb vector.

### Cell culture

A549 cells stably expressing ^3xFLAG^NS1-BP were generated as previously described (15). DLD-1-^HA-AID^GANP cells were generated as previously described (26). Human lung adenocarcinoma epithelial cells (A549 CRN-CCL-185) were obtained from American Type Culture Collection, which are validated using short tandem repeat profiling. A549 NS1-BP^+/+^ and NS1-BP^−/−^ cells were generated as previously described (28). All cells were cultured in high-glucose Dulbecco's modified Eagle's medium (Gibco) medium with 10% fetal bovine serum (Atlas) and Pen/Strep antibiotics. Cells were cultured at 37  °C in the presence of 5% CO_2_. Cells are monthly checked for *mycoplasma* contamination.

### Viruses

All virus work was performed as per CDC guidelines for biosafety level 2. IAVs were generated as we previously described (12).

### Infection

A549 cells were washed with infection medium [Eagle's minimum essential medium from American Type Culture Collection, 10 mM Hepes (Gibco), 0.125% bovine serum albumin (Gibco), 0.5 μg/ml TPCK trypsin (Worthington Biomedical Corporation)] and incubated with IAVs (PR8-TX-NS NS1^WT^ or PR8-TX-NS NS1^F^^103A/F138A^) at multiplicity of infection 2 for 1 h. Cells were then washed with Eagle's minimum essential medium infection medium and incubated in the same medium for 8 h.

### Subcellular fractionation, RNA purification, and qPCR

Subcellular fractionation to obtain nuclear and cytoplasmic fractions of NS1-BP^+/+^ and NS1-BP^−/−^ cells was performed as we previously described (24). RNA purification and qPCR was performed as we reported (15).

### Protein expression and purification

In Fig. 3, *D* and *E*, recombinant NS1-BP full length and its domains were expressed and purified as we previously described (15). NXF1-NXT1 full length and NXF1 domains were purified and expressed as we previously reported (12).

In Fig. 7*B*, NXF1–NXT1 complex was purified as previously described (12). GFP-NS1 and NS1-BP were expressed in *Escherichia coli* Rosetta cells (Sigma-Aldrich). IPTG at 0.5 mM was used to induce protein expression at 20 °C during overnight incubation. Cells were lysed in a buffer containing 50 mM Tris (pH 8.0), 300 mM NaCl, 20 mM imidazole, 0.5 mM tris(2-carboxyethyl)phosphine (TCEP), 0.2 mM 4-(2-aminoethyl)benzenesulfonyl fluoride hydrochloride, and 2 mg/l aprotinin. GFP-NS1 and NS1-BP were pulled down using Ni Sepharose resin (Cytiva). Samples were further purified using a Superdex 200 column (Cytiva) equilibrated with 10 mM Tris (pH 8.0), 300 mM NaCl, and 0.5 mM TCEP. GFP-NS1 and NS1-BP were stored in 10% glycerol. All proteins were concentrated, aliquoted, flash-frozen in liquid nitrogen, and stored at −80 °C.

### Electrophilic mobility shift assay

GFP-NS1 (0.1 μM) was mixed with NXF1-NXT1 (3 μM) and increasing concentrations of NS1-BP in a buffer containing 10 mM Tris (pH 8.0), 150 mM NaCl, 0.5 mM TCEP, 0.5 mg/ml bovine serum albumin and 8% glycerol. The mixtures were incubated on ice for 30 min. Samples were separated on a 5% native PAGE gel that was made and run in a buffer containing 45 mM Tris and 16 mM boric acid (adjusted to increase the pH to allow NS1-NXF1-NXT1 to enter the gel). GFP-NS1 was visualized using a ChemiDoc MP Imaging System (Bio-Rad). The experiments were conducted three times independently.

### Immunoprecipitation

All antibodies were validated by knocking down or knocking out the protein of interest. In Fig. 3*B*, A549 cells grown in 10 cm plates were noninfected or infected with PR8-TX-NS NS1^WT^ virus or PR8-TX-NS NS1^F103A/F138A^ virus for 6 h as described in Zhang *et al.* (12). Cells were lysed for 30 min at 4  °C while vortexing every 5 min in 50 mM Tris, pH 7.5, 150 mM NaCl, 1% octylphenoxypolyethoxyethanol (IGEPAL CA-630), 0.1 mM Na3VO4, 1 mM NaF, 1 mM DTT, 1 mM EDTA, 1 mM PMSF, 1 × complete protease inhibitor cocktail, and 10% glycerol. Cell lysates were centrifuged at 13,000*g* for 10 min to remove cellular debris. The supernatant was applied to protein G beads bound to either mouse IgG (sc-2025, Santa Cruz Biotechnology) or anti-TAP(NXF1) mouse mAb (T1076, Sigma-Aldrich Co. LLC) and incubated for 4 h at 4  °C. After five washes with lysis buffer, the proteins were eluted with 2 × sample buffer [125 mM tris (pH 6.8), 10% glycerol, 2% SDS, and 0.0025% bromophenol blue] and subjected to 8% SDS–PAGE followed by Western blot to detect NS1-BP and NS1. NS1-BP was detected with a rabbit anti-NS1-BP antibody (A302-879A, Bethyl Laboratories, Fortis Life Sciences), and NS1 was detected with a rabbit anti-NS1 antibody, a gift from J.A. Richt (National Animal Disease Center) (37).

In Fig. 3*C*, 293T cells were transfected with 3XFLAG vector control or 3XFLAG-NS1-BP using TransIT-X2 (Mirus Bio LCC, MIR6000). Cells lysates were prepared as described for Fig. 2*A*. The cleared lysates were applied to anti-FLAG M2 magnetic beads (M8823, Sigma-Aldrich Co. LLC) in the presence of RNasin (1 U/μl) or RNase A (10 μg/ml) for binding overnight at 4 °C. Beads were washed five times with lysis buffer at 4 °C. Then, proteins were eluted using 3×Flag peptide (APE × BIO, A6001). The eluted fractions were mixed with 2 × sample buffer [125 mM tris (pH 6.8), 10% glycerol, 2% SDS, and 0.0025% bromophenol blue] and subjected to 8% SDS-PAGE followed by Western blot. NXF1 was detected by a mouse mAb (T1076, Sigma-Aldrich Co. LLC). NS1-BP was detected with anti-Flag mouse mAb (Sigma Aldrich F1804). GANP was detected with a rabbit polyclonal antibody (Bethyl Laboratories cat# A303-127A). NUP98 was detected with a rabbit polyclonal antibody previously described (38).

### GST pull-down assays

For Fig. 3*D*, GSH-bead immobilized purified GST protein or GST-NS1-BP protein or GST-NS1-BP protein domains were incubated with 1 μM His–NXF1•NXT1 purified protein in the binding buffer (20 mM Tris, 150 mM NaCl, 1 mM DTT, 1 mM EDTA, pH 7.5) at room temperature for 30 min. Beads were pelleted by centrifugation at 5000 rpm for 5 min and washed 5x with 1 ml of binding buffer. Proteins remaining on the resin were extracted by sample buffer, resolved in SDS-PAGE, and then detected by Western blot using anti-TAP(NXF1) mouse mAb (T1076, Sigma-Aldrich Co. LLC).

For Fig. 3*E*, GSH-bead immobilized purified GST protein or GST–NXT1•His–NXF1 proteins or GST–NXF1 domains were incubated with 1 μM His–NS1-BP purified protein. Pull-down elutes were subjected to SDS-PAGE, and NS1-BP was detected with anti-NS1-BP antibody (A302–879A, Bethyl Laboratories, Fortis Life Sciences).

### *In vitro* binding and competition assays

Two micromolar of GST–NXT1•His–NXF1 recombinant protein complex or GST protein incubated with 2 μM of MBP-NS1 purified protein in the presence of 10 μl dry volume of GSH beads were incubated for 1 h at room temperature (RT). Immobilized GST–NXT1•His–NXF1•MBP-NS1 complex or GST was washed with Tris-Buffered Saline with Tween 20 (TBS-T; T1688, Teknova) once, and increasing amounts (2 μM, 4 μM and 8 μM) of His-NS1-BP were added to the pre-washed immobilized complexes for competition assay. Assay solution was incubated at RT for 1 h, then GSH complex–bounded GSH beads were washed with TBS-T for four times. Proteins were eluted with 2 × sample buffer and subjected to 8% SDS–PAGE, followed by Western blot using the antibodies described in the sections above.

Myc-NUP98 protein (UniProtKB accession number P52948) and Flag–NUP153 (UniProtKB accession number P49790) were generated by using the coupled rabbit reticulocyte lysate transcription–translation system according to the manufacturer’s instructions (Promega, L4610). 2 μM GST–NXT1•His–NXF1 recombinant protein complex or GST protein, 25 μl of either NUP98 or NUP153 transcription–translation reaction, and 10 μl dry volume of GSH beads were incubated for an hour at RT. Immobilized GST–NXT1•His–NXF1•NUP complex or GST was washed with TBS-T once, and increasing amounts (2 μM and 8 μM) of His-NS1-BP were added to the pre-washed immobilized complexes for competition assay. Assay solution was incubated at RT for 1 h, and then GSH complex–bounded GSH beads were washed with TBS-T four times. The proteins were eluted with 2 × sample buffer and subjected to 8% SDS–PAGE followed by Western blot. NUP98 was detected with a rabbit polyclonal antibody previously described (38) and NUP153 was detected with anti-Flag mouse monoclonal antibody (Sigma Aldrich F1804).

### Single-molecule RNA FISH

smRNA FISH for M, NS, HA, NA, NP, PB1, and PB2 mRNAs was performed as previously described (13). We have previously described the M, NS, HA, and NP Stellaris RNA FISH probes sequences (13, 24, 28). PB1 and PB2 Stellaris RNA FISH probes sequences are available in the Supporting information file. For detection of PA mRNA, ViewRNA Cell Plus Assay Kit was used (Invitrogen, 88-19000-99). ViewRNA Type 1 Probe Set (Invitrogen, Thermo Fisher Scientific) was custom designed by online tool (https://www.thermofisher.com/order/custom-oligo/brancheddna). NS1-BP^+/+^ or NS1-BP^−/−^ A549 cells were seeded at a density of 30,000 cells per well in an 8-well chamber slide. The next day, cells were infected with IAV (A/WSN/33) at multiplicity of infection 2. After 8 h of infection, cells were fixed according to the manufacturer’s instructions. The manufacturer’s protocol for samples processed on 8-well chamber slides was followed with additional modifications: A hybridization oven (HYBAID, HS9360) at 40 °C was used for all steps requiring 40 °C. Slides were incubated over a humidified tissue with sterile deionized water in a tight-fit closed box for all steps requiring a humidified staining tray. Probes were used at a 1:100 dilution. Prolong Gold Antifade Reagent (P36930, Invitrogen, Thermo Fisher Scientific) was used for mounting. Samples were stored at RT in the dark to cure the mounting reagent before acquisition.

### PLA combined with immunofluorescence microscopy

A549 cells were fixed in 4% formaldehyde in PBS at room temperature for 15 min and permeabilized with 0.2% Triton X-100 in PBS for 5 min at 4 °C. Samples were then rinsed briefly with PBS and incubated with the Duolink blocking *solution* (Duolink In Situ Red Starter Mouse/Rabbit kit, DUO9210, Sigma-Aldrich Co. LLC) in a preheated humidity chamber for 1 h at 37 °C. Primary antibody cocktail [anti-NS1-BP rabbit polyclonal antibody (A302-879A, Bethyl Laboratories, Inc., diluted at 1:100) and anti-TAP(NXF1) mouse mAb (T1076, Sigma-Aldrich Co. LLC, diluted at 1:250)] was diluted in the *Duolink antibody diluent* solution and added to the cells, followed by incubation for 1 h at room temperature. Then the PLA assay was completed according to the manufacturer's instructions. Next, samples were incubated with Hoechst 33,342 (1.5 ug/ml, Molecular Probes) for nuclear counterstaining, then briefly washed with PBS for 5 min, and Antifade Gold mounting media was used for mounting. PLA signal specificity was assessed by using primary antibody cocktails as following: (1) double-minus (no primary antibody), (2) NS1-BP antibody alone, and (3) NXF1 antibody alone. Images were acquired with a Zeiss Axiovert 200 M automated microscope using a 63X Plan-APOCHROMAT lens (1.4 numerical aperture) and captured by a Hamamatsu ORCA-spark CMOS monochrome camera. Cells were visualized sequentially for PLA signals, recognized as red fluorescent spots using a tetramethylrhodamine filter and DNA fluorescence ET434/32 filter, respectively, using a step size of 0.5 μm (total z size 10 μm). Acquired z stack images were blind deconvolved using Autoquant X (Media Cybernetics).

### Imaris-assisted image analysis for PLA assay

The Imaris software package Cells module (Bitplane, Version 9.8.2; https://imaris.oxinst.com/versions/9-8) was used for visualization and quantification in 3D isometric rendering images of the nucleus (chromatin) and PLA signal of the NXF1/NS1-BP complex display of confocal z stack fluorescence. First, nuclei (Hoechst 33342) were segmented using an automated threshold (based on the intensity distribution histogram) in 434/32 nm channel by Imaris surface tool. Identified nuclei populations were filtered to remove large nuclei aggregates (upper nuclei volume threshold and lower threshold to manual remove fragments of nuclei at the edges of the stack) using the Imaris surface function. Next, PLA signals were identified in tetramethylrhodamine channel using Imaris spot tool. The seed spot size used was 0.2 to 0.35 mm. For objective PLA spot center identification, automatic thresholds were used to filter raw spot quality. After identification of all PLA spots in the cell, the nuclear PLA spots were segmented, setting spot filter function to the “shortest distance to nucleus,” upper-threshold to 0, and turning the lower-threshold off, remaining PLA spots were assigned as cytoplasmic signal. 3D volume blend rendering projection image was created by giving chromatin and PLA voxels in the scene 40% and 100% opacity, respectively, to suppress and enhance their influence in the rendered image without interfering with the original structure of the data. Brightness and contrast were linearly enhanced using Adobe Photoshop's Level tool. Statistical analysis was carried out using the one-way ANOVA with a Tukey post test (Prism 9, GraphPad; https://www.graphpad.com).

## Data availability

The data generated in this study are within the article or in the Supporting information file.

## Supporting information

This article contains supporting information.

## Conflict of interest

The A. G.-S. laboratory has received research support from GSK, Pfizer, Senhwa Biosciences, Kenall Manufacturing, Blade Therapeutics, Avimex, Johnson & Johnson, Dynavax, 7Hills Pharma, Pharmamar, ImmunityBio, Accurius, Nanocomposix, Hexamer, N-fold LLC, Model Medicines, Atea Pharma, Applied Biological Laboratories, and Merck, outside of the reported work. A. G.-S. has consulting agreements for the following companies involving cash and/or stock: Castlevax, Amovir, Vivaldi Biosciences, Contrafect, 7Hills Pharma, Avimex, Pagoda, Accurius, Esperovax, Applied Biological Laboratories, Pharmamar, CureLab Oncology, CureLab Veterinary, Synairgen, Paratus, Pfizer, and Prosetta, outside of the reported work. A. G.-S. has been an invited speaker in meeting events organized by Seqirus, Janssen, Abbott, Astrazeneca, and Novavax. A. G.-S. is the inventor on patents and patent applications on the use of antivirals and vaccines for the treatment and prevention of virus infections and cancer, owned by the Icahn School of Medicine at Mount Sinai, New York, outside of the reported work. B. M. A. F. holds the Ruth S. Harrell Professorship in Medical Research. The other authors declare that they have no conflicts of interest with the contents of this article.
