# Supplementary material for: Cellular NS1-BP protein interacts with the mRNA export receptor NXF1 to mediate nuclear export of influenza virus M mRNAs
Source: J Biol Chem. 2024 Oct 9;300(11):107871. doi: 10.1016/j.jbc.2024.107871 (PMC11570952; doi:10.1016/j.jbc.2024.107871)
Supplement: Supporting information [file mmc1.pdf]

Supporting Information

Cellular NS1-BP Protein Interacts with the mRNA Export Receptor NXF1 to Mediate Nuclear Export of Influenza Virus M mRNAs

Ke Zhang[1,2*], Tolga Cagatay[1*], Dongqi Xie[1], Alexia E. Angelos[3], Serena Cornelius[1], Vasilisa Aksenova[4], Sadaf Aslam[5], Zhiyu He[2], Matthew Esparza[1], Ashley Vazhavilla[1], Mary Dasso[4], Adolfo García-Sastre[5,6,7,8,9,10], Yi Ren[3], Beatriz M. A. Fontoura[1§]

[1]Department of Cell Biology, University of Texas Southwestern Medical Center, Dallas, Texas 75390, USA

[2]Shanghai Institute of Immunity and Infection, Chinese Academy of Sciences, Shanghai 200031, China

[3]Department of Biochemistry, Center for Structural Biology, Vanderbilt University School of Medicine, Nashville, TN 37232, USA

[4]Division of Molecular and Cellular Biology, National Institute of Child Health and Human Development, National Institutes of Health, Bethesda, MD 20892, USA

[5]Department of Microbiology, Icahn School of Medicine at Mount Sinai, New York, NY 10029, USA

[6]Global Health and Emerging Pathogens Institute, Icahn School of Medicine at Mount Sinai, New York, NY 10029, USA

[7]Department of Medicine, Division of Infectious Diseases, Icahn School of Medicine at Mount Sinai, New York, NY 10029, USA

[8]Department of Pathology, Molecular and Cell-Based Medicine, Icahn School of Medicine at Mount Sinai, New York, NY 10029, USA

[9]The Tisch Cancer Institute, Icahn School of Medicine at Mount Sinai, New York, NY 10029, USA

[10]The Icahn Genomics Institute, Icahn School of Medicine at Mount Sinai, New York, NY 10029, USA

*These authors equally contributed to the manuscript.
§Corresponding Author: Beatriz M. A. Fontoura
(Beatriz.Fontoura@UTSouthwestern.edu)

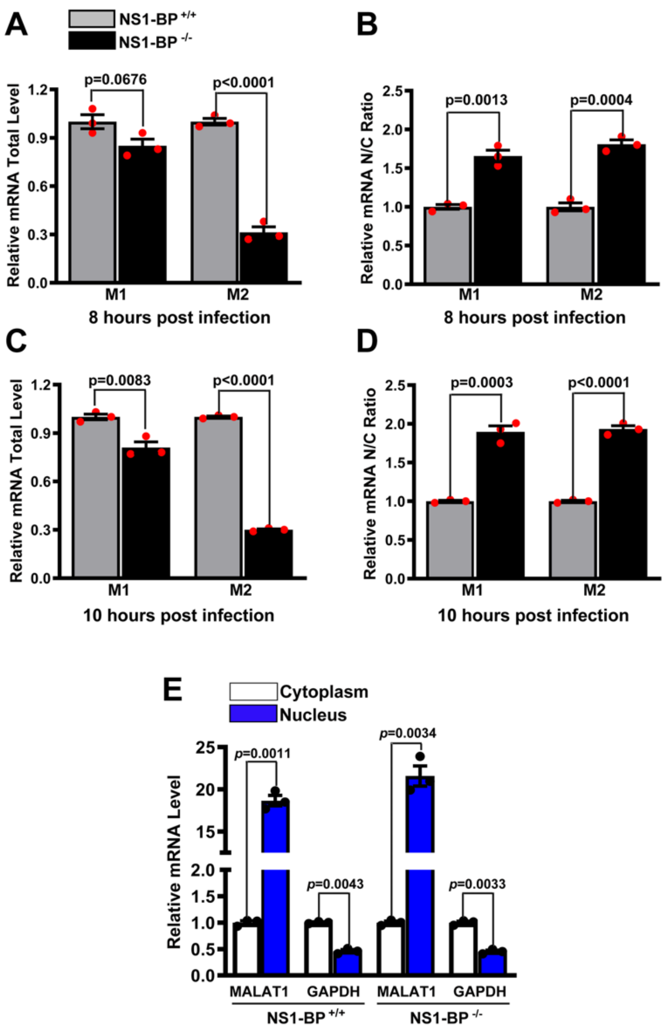

Figure S1. NS1-BP knockout cells show down-regulation of influenza virus M1 to M2 mRNA splicing and significant retention of both mRNAs in the nucleus. A-E NS1-BP$^{+/+}$ or NS1-BP$^{-/-}$ cells were infected with A/WSN/33 for 8 h or 10 h. ***A,C,*** RNA was purified from total cell lysates and qPCR was performed to determine the levels of M1 and M2 mRNAs at 8h and 10 h. ***B,D,*** RNA purified from nuclear and cytoplasmic fractions were subjected to qPCR to determine the levels of M1 and M2 mRNAs in each fraction as well as the levels of MALAT1 (control for nuclear fraction) and GAPDH (control for cytoplasmic fraction) shown in ***E***. *n=3; ***p<0.001.*

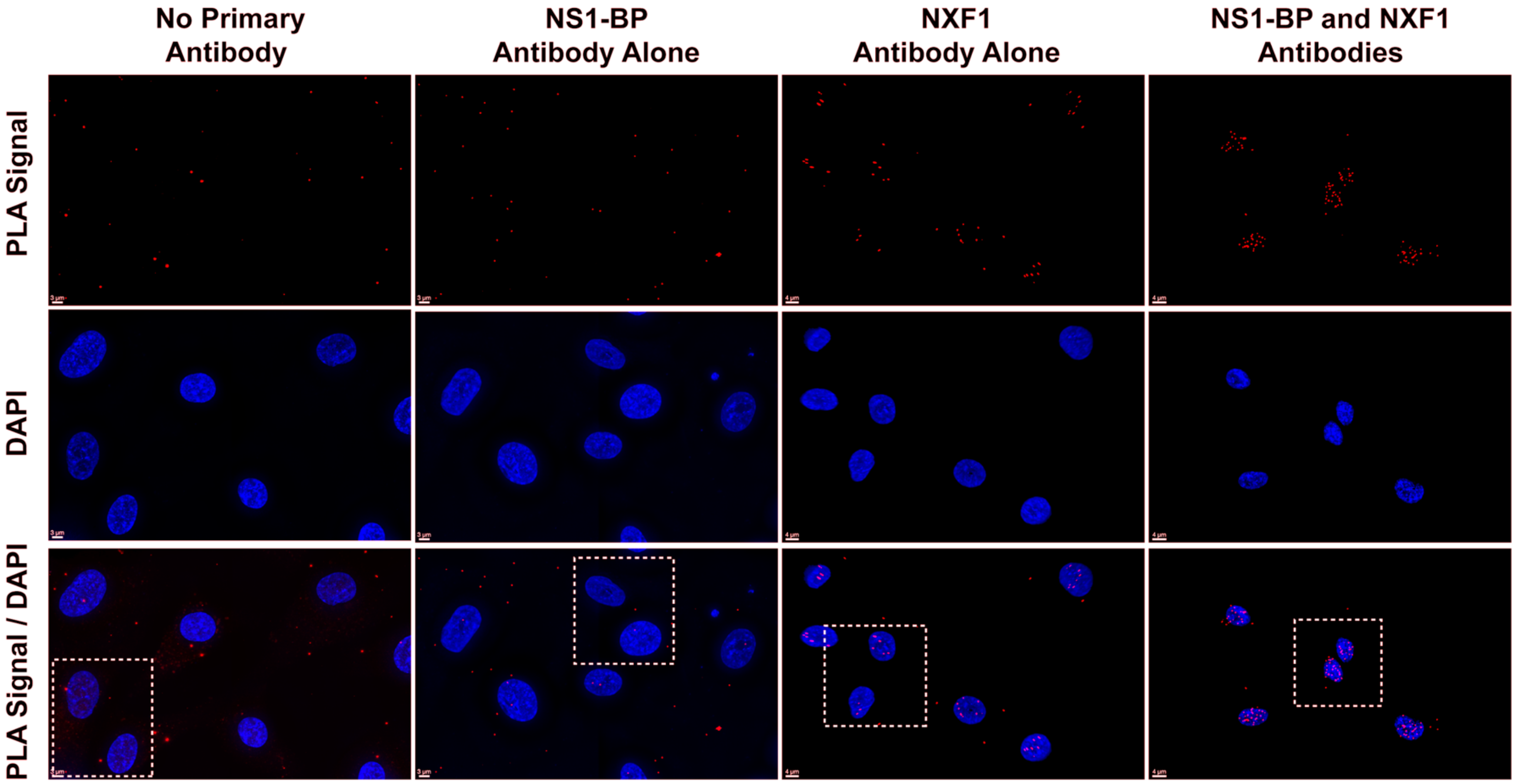

Figure S2. Field of view of the images shown in Figure 4. NS1-BP interaction with NXF1 occurs in the nucleus. A549 cells were subjected to Proximity Ligation Assay (PLA) to detect the interaction between NS1-BP and NXF1 proteins *in situ*. The interaction by PLA is detected by fluorescent probes (red dots; λem = 624 nm, TRITC filter). Hoechst staining labels the nuclei (blue). Dashed squares are the regions shown in Figure 4. Quantification is shown in Figure 4.

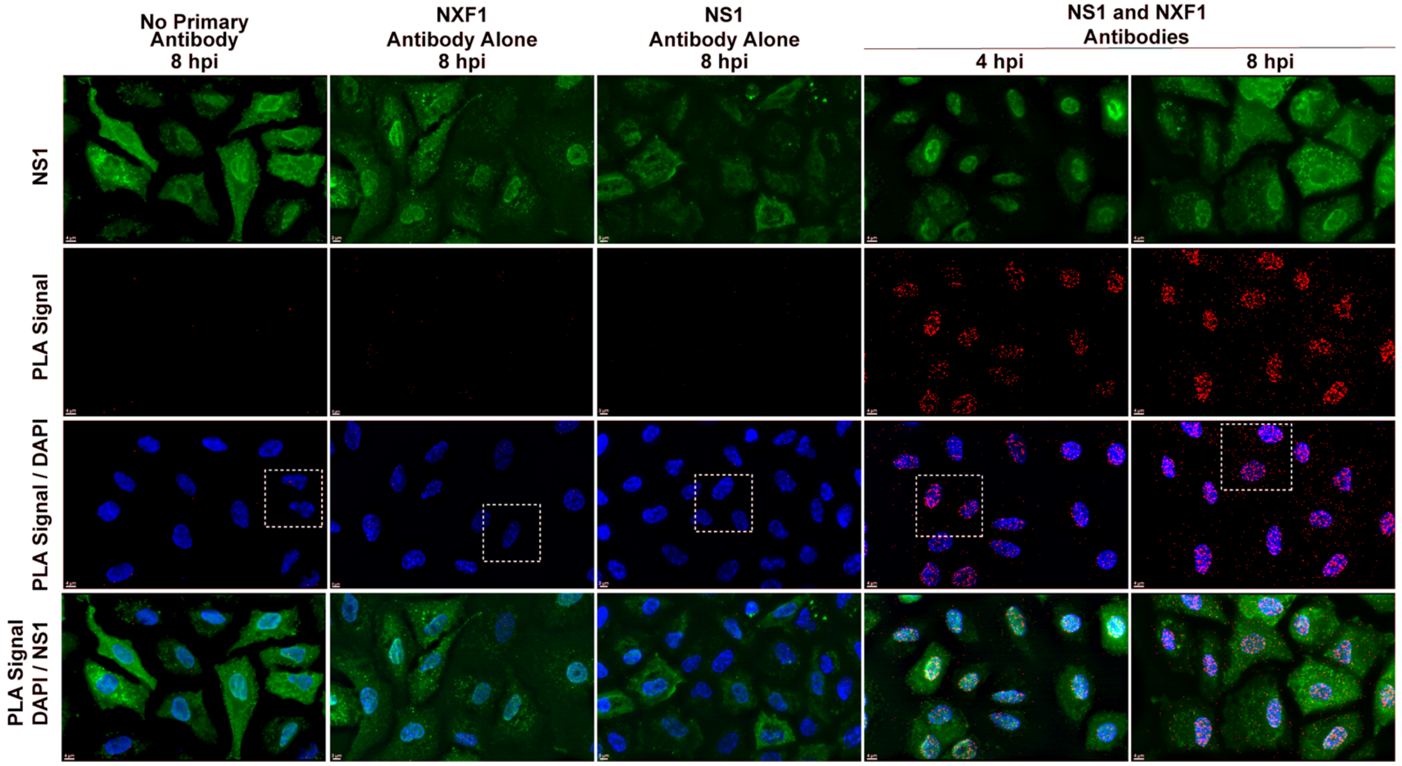

Figure S3. Field of view of the images shown in Figure 5. Temporospatial dynamics of NS1 and NXF1 interaction during infection. A549 cells infected with influenza virus were subjected to PLA to detect the interaction between NS1 and NXF1 proteins *in situ* at 4hpi (hours post infection) or 8hpi. The interaction by PLA is detected by fluorescent probes (red dots; λem = 624 nm, TRITC filter). Hoechst staining labels the nuclei (blue). Quantification is shown in Figure 5.

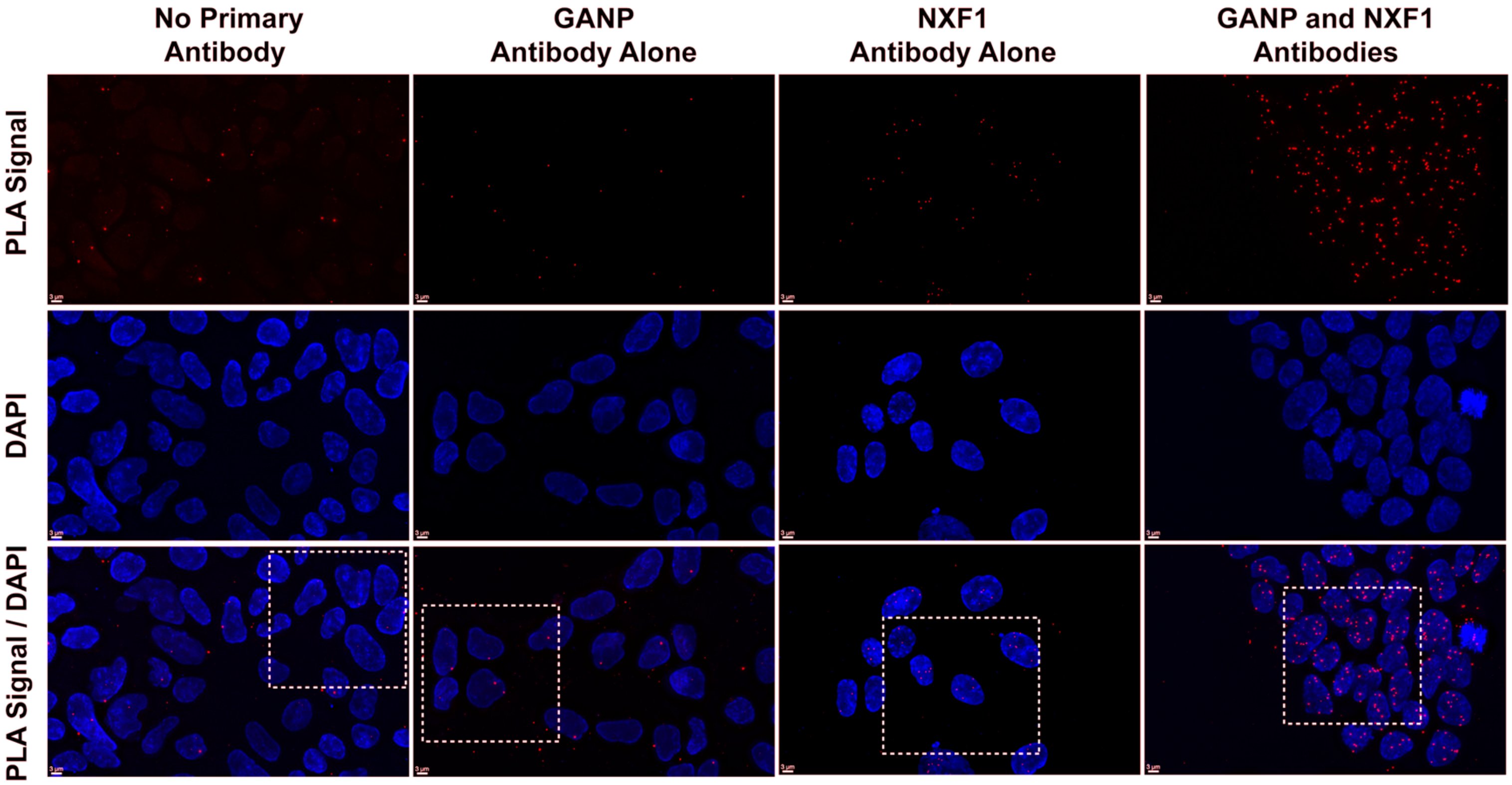

Figure S4. Field of view of the images shown in Figure 6. GANP and NXF1 interaction is detected in the nucleoplasm and in the nuclear periphery. DLD1- $^{HA\text{-}AID}$GANP cells were subjected to PLA to detect the interaction between GANP and NXF1 proteins *in situ*. The interaction by PLA is detected by fluorescent probes (red dots; λem = 624 nm, TRITC filter). Hoechst staining labels the nuclei (blue). Quantification is shown in Figure 6.

ViewRNA IAV PA bDNA probe

Probe #	Geneset Name	Sequence (5'-->3')
1	Influenza A Virus IAV PA	ttgtcgcacaaaatcttccatt
2	Influenza A Virus IAV PA	gacaatcatcggattgaagca
3	Influenza A Virus IAV PA	tgccttttccgcaagctc
4	Influenza A Virus IAV PA	caggtcctctccatactctttcat
5	Influenza A Virus IAV PA	gcaaatttgtttgtttcgatttt
6	Influenza A Virus IAV PA	cttccaagtgagtgcatattgct
7	Influenza A Virus IAV PA	tgaaaatctgaatacatgaagcaca
8	Influenza A Virus IAV PA	cgccttgctcatcgatgaag
9	Influenza A Virus IAV PA	cgccaagttctacgactattgact
10	Influenza A Virus IAV PA	tgcttcaaaagtgcatttggat
11	Influenza A Virus IAV PA	cttccctcgattatttcaaatctg
12	Influenza A Virus IAV PA	gtccaggctattgtgcgatct
13	Influenza A Virus IAV PA	ctgtagtgttgcaaatactgtttattact
14	Influenza A Virus IAV PA	agaaactttggtttctcagccc
15	Influenza A Virus IAV PA	ttcttcttgtaatcatacaaatctggt
16	Influenza A Virus IAV PA	tgttactccaatttcgatgaatcta
17	Influenza A Virus IAV PA	ccagatagtatatgtgaacttctctcct
18	Influenza A Virus IAV PA	tcagatttaattttattggcctttt
19	Influenza A Virus IAV PA	tgagaaaatgtggatgtgtgtcttc
20	Influenza A Virus IAV PA	gccatttcctccccagtgaa
21	Influenza A Virus IAV PA	gagagtgtagtcggcctttgtg
22	Influenza A Virus IAV PA	tcctagccctgctttcttcatc
23	Influenza A Virus IAV PA	atggtgaatagcctggttttga
24	Influenza A Virus IAV PA	cctctgctagccatttcttgtctt
25	Influenza A Virus IAV PA	tgacgaaaggaatcccagagg
26	Influenza A Virus IAV PA	gtctcttcgcctctctcggac
27	Influenza A Virus IAV PA	ctgtgatttcaaatctttcttcaatt
28	Influenza A Virus IAV PA	gcaagcttgcgcattgttc
29	Influenza A Virus IAV PA	ggcgggagactttggtcg
30	Influenza A Virus IAV PA	atttttcaaggctggagaagttt
31	Influenza A Virus IAV PA	gaatccatccacataggctctaa
32	Influenza A Virus IAV PA	ctcaatgtagccgttcggttc
33	Influenza A Virus IAV PA	ggacatttgagaaagcttgcc

34	Influenza A Virus IAV PA	ggttcaattctagcatttacttcttt
35	Influenza A Virus IAV PA	gtcgtggtgttgatttcaaaaaa
36	Influenza A Virus IAV PA	gcccatccggaagtctaagtg
37	Influenza A Virus IAV PA	ggaccgctgagaacagggag
38	Influenza A Virus IAV PA	ggcatccatcagcaggaattt
39	Influenza A Virus IAV PA	tgggtcctcaatgcttaattttaa
40	Influenza A Virus IAV PA	ggtatcccctctccctcatgact
41	Influenza A Virus IAV PA	atgcatttgattgcatcatatagc

IAV NA Stellaris Probe

Probe #	Probe (5'-> 3')
1	atttaaactcctgctttcgc
2	cccaatggttattattttct
3	ttccgactaccatacagatt
4	cctatttgcaatattaggct
5	ccggtttgaattgaatggct
6	gcatattccagtatggtttt
7	gcccagcaacaactttatag
8	ttgccggttaatatcactga
9	ccacggatgggacaaagaga
10	ttgtctttgctgtgtatagc
11	ctcctttggaaccaattctt
12	ctgcattccaagtgagaaca
13	ggcgccttgagtcagaaaaa
14	ccttgaatgcttgtcattca
15	ataagggcttctgtccttaa
16	cgacagggcagctcattaag
17	ttgaattgtacggggacgga
18	tgaccaagcaaccgattcaa
19	cattccatcatgacatgcac
20	ggaccagaaattccgattgt
21	ttttaatacagccactgctc
22	ggtttcagttattatgcggt
23	tattcttcctccaactttttt
24	ttcagactcttgtgttctca
25	catgaaccatttacacaggt
26	gccatcggtcattatggtaa

27	aaattttgtacgaggccagc
28	atcgatttagtaaccttccc
29	cctcgtagtgagaattaggt
30	cggtatcagggtaacaggaa
31	caattgtctctgcacacaca
32	tctaggttttggtcgaagga
33	cactgcagatgtatcctatt
34	cgcgggttgtcaccgaaaac
35	aaaatccctttactccgttt
36	acaccattgccatacttata
37	cacttttagtccttcctatc
38	tcaaacccatgtctggaact
39	atccattaggatcccaaatc
40	aacctactatcagtctctgt
41	ccacaacatcttgtctcata
42	taccctgaccgattagttat
43	ctgttagctcaggatgttga
44	caaggcctcatacagtctag
45	cctgattaattcaacccaga
46	aaatgatgctcccactagtc
47	gtatcactattcacaccaca
48	gtcaatggtgaacggcaact

IAV PB1 Stellaris Probe

**Probe #**	**Probe (5'-> 3')**
1	gcttatagcattttgtgctg
2	aaggagggtctccagtataa
3	cctttctgagtactgatgtg
4	catcaatcgggttgagttgc
5	acttggttcattgtcttctg
6	ccattgcttccaatacacaa
7	gtttcaagacacgaggtctc
8	cactcgtgtttgctgaacaa
9	tttagagtccagtcataggt
10	ttctattgtgttggccaatg

11	tgaggccatttgatctgaac
12	aaggaagtctatgagccttc
13	tcattgactccattacatcc
14	gtcatattgtctctcactcg
15	caatctctgcttccttttac
16	tgccctaattagataactcc
17	tctttggtcattgtgttcag
18	agtatacaaaccccccttatt
19	cacatatactccttgctagt
20	aactggcaatcctgattgtt
21	tccttacaacatttgccaac
22	tgtcctgagaattggtcatc
23	atttggtgttatctccagtg
24	aaacatccgagggttctgat
25	acatttctgaaccattcggg
26	ctcgccattttgtttgagaa
27	ttttcatactcttgctctca
28	aatcgatgcttgctagcatt
29	atgcagtcccatctattaag
30	catattgaacatgcccatca
31	tgtccaagattcaggatgga
32	gtaagtagtcttggtgtgtc
33	gaagattgaagaccatccca
34	gggtgcattcacaatcagag
35	ctccatgctgaaattggcaa
36	aactccaatactcatgtccg
37	ttgctggaccaagatcattg
38	gtacgtgtacctgtaatctt
39	ttgtataaatttgggcctcc
40	agcagacttcaggaatgtgg
41	tcatccattaattcccattt
42	tcagtgggttgcataaacgc
43	agcatcatactccatgtttt
44	aagtattcctctttggcttg
45	tgcagcacttttggtacatt
46	tggtcttctgtatgaactgc
47	tcaattcgggctctggaaac

48	gctcttcaatggtggaacag

IAV PB2 Stellaris Probe

Probe #	Probe (5'-> 3')
1	cggtggtttttgtgagtatc
2	ctcttgtctgctgtaattgg
3	tctcaggaatcatttccgtt
4	tactccataaagtttgtccc
5	gaactgtacttgtcactggt
6	tccatgttttaacctttcga
7	ttctaaaatggacagggcca
8	tctgcatgaccaggatttat
9	ttccatgattacatcctgtg
10	cccacttcgttagggaaaac
11	tgcgattccgatgttagtat
12	ctgggaggaatctcgttttg
13	ttccttgggtcaaatgcaac
14	ctagtggatctgctgatact
15	ggatgtttaccatccttatt
16	ttgcaaatatccacggcttg
17	gatgagctaattctcagtcc
18	gatccgcttgttctcttaaa
19	ctcatgcactcttatcttca
20	ccaaccattgtgaactcttc
21	atcagctgaatcaatctcct
22	atggccacaattattgcttc
23	gacgaaattcaggtcacctc
24	ttggtgcatgggattcaatc
25	cctttgcatccttctgaaaa
26	tggattcaattccccaattt
27	aaacggtcaatgctcaccac
28	ctcgggagacagtagtacat
29	gttattgtcagtttctctgt
30	ccacatcattgacgatgagt
31	acagcattgtaggattctgg

32	tctaacggcctttggaacta
33	tctcacaaacccactgtatt
34	tcaaatgtcccaagcacatc
35	gctgcgaagggaagaagttt
36	agaactgcgttctactttgc
37	aattgccccttacaagtatt
38	agtggtcttgttgtagttga
39	ctgggtcttcagttaaaggg
40	cagaactgcggactcaactc
41	ctttgcccagaatgaggaat
42	cttaatgctggtccatatct
43	caaggttgctcagttcattt
44	ttgcccaattagcacattag
45	cgtttcattaccaacaccac
46	aagtatgctagagttccgtt
47	aattgatggccatccgaatt
